# Semi-quantitative proteomics of mammalian cells upon short-term exposure to non-ionizing electromagnetic fields

**DOI:** 10.1371/journal.pone.0170762

**Published:** 2017-02-24

**Authors:** Arnold Kuzniar, Charlie Laffeber, Berina Eppink, Karel Bezstarosti, Dick Dekkers, Henri Woelders, A. Peter M. Zwamborn, Jeroen Demmers, Joyce H. G. Lebbink, Roland Kanaar

**Affiliations:** 1 Department of Molecular Genetics, Cancer Genomics Netherlands, Erasmus University Medical Center, Rotterdam, The Netherlands; 2 Netherlands eScience Center, Amsterdam, The Netherlands; 3 Proteomics Center, Erasmus University Medical Center, Rotterdam, The Netherlands; 4 Wageningen Livestock Research, Wageningen, The Netherlands; 5 TNO, The Hague, The Netherlands; 6 Netherlands Proteomics Center, Rotterdam, The Netherlands; 7 Department of Radiation Oncology, Erasmus University Medical Center, Rotterdam, The Netherlands; Consiglio Nazionale delle Ricerche, ITALY

## Abstract

The potential effects of non-ionizing electromagnetic fields (EMFs), such as those emitted by power-lines (in extremely low frequency range), mobile cellular systems and wireless networking devices (in radio frequency range) on human health have been intensively researched and debated. However, how exposure to these EMFs may lead to biological changes underlying possible health effects is still unclear. To reveal EMF-induced molecular changes, unbiased experiments (without *a priori* focusing on specific biological processes) with sensitive readouts are required. We present the first proteome-wide semi-quantitative mass spectrometry analysis of human fibroblasts, osteosarcomas and mouse embryonic stem cells exposed to three types of non-ionizing EMFs (ELF 50 Hz, UMTS 2.1 GHz and WiFi 5.8 GHz). We performed controlled *in vitro* EMF exposures of metabolically labeled mammalian cells followed by reliable statistical analyses of differential protein- and pathway-level regulations using an array of established bioinformatics methods. Our results indicate that less than 1% of the quantitated human or mouse proteome responds to the EMFs by small changes in protein abundance. Further network-based analysis of the differentially regulated proteins did not detect significantly perturbed cellular processes or pathways in human and mouse cells in response to ELF, UMTS or WiFi exposure. In conclusion, our extensive bioinformatics analyses of semi-quantitative mass spectrometry data do not support the notion that the short-time exposures to non-ionizing EMFs have a consistent biologically significant bearing on mammalian cells in culture.

## Introduction

Modern society is becoming more and more dependent on electrical power to fuel a wide range of equipments including communication devices. This has resulted in an increase of exposure to extremely low frequency (ELF) and radio frequency (RF) electromagnetic fields (EMFs). There has been a long-running debate on the health effect of these non-ionizing EMFs [1]. However, prior to formulating useful and testable hypotheses on the potential adverse or beneficial influence of EMF exposure on human health it is imperative that the biological effects on the cells are detected unambiguously [2–5].

Cells are the building blocks of organs and organisms and in order to survive they have evolved the ability to respond to a wide range of stimuli presented from the environment. Cellular responses are mediated through molecular signaling pathways, which consist of receptors for the signal that activate transducers, which in turn stimulate affecters that illicit the appropriate molecular response [6]. Classic examples of such responses to environmental cues are growth factor signaling and the DNA damage response. Specifically, response to growth factors occurs through receptor molecules on the cell surface that through conformational changes induce post-translational modification of proteins in the cytoplasm. This eventually results in the activation of nuclear transcription factors that turn on/off the genes whose products (or their absence) mount the appropriate cellular response. In case of the DNA damage response, nuclear DNA is the ‘receptor’ because when its integrity is disturbed by DNA damaging agents, such as ionizing radiation or tobacco smoke, it triggers cell cycle arrest and downstream biological effects such as apoptosis or repair of the DNA lesions [7–9].

By triggering a cellular response non-ionizing EMFs could influence health. However, currently it is unclear if and how cells can sense these EMFs. Cellular sensing of EMFs requires changes in the molecular constituents of the cell in order to activate a signaling pathway. As of to date, no unambiguous and reproducible molecular changes including a perturbed biological pathway(s) have been detected in cells exposed to ELF- or RF-EMFs. With advances in transcriptomics, several studies analyzing changes in gene expression in bacteria, yeasts, neurons, white blood cells, keratinocytes and cancer cells in response to ELF or RF exposure have been published to date [10–17]. In addition, the proteomes of human monocytes, lymphoblastoid B cells and endothelial cells in response to RF exposure have also been analyzed [18–20].

Taken together, these studies did not identify common, consistently affected molecules and/or cellular pathways. Therefore, the inevitable conclusion is that the effects on molecular changes induced by these EMFs are probably subtle, otherwise a consistent signaling pathway(s) would already have been identified, for example, as in case of the cellular response to ionizing radiation [7]. Clearly, if a cellular response is to be detected, the most sensitive and specific methods have to be applied, otherwise it will be very unlikely that an EMF signature can be identified, in particular given the stochastic variation in the intracellular ratios of molecular constituents that is characteristic of biological systems [21].

In this study, we have taken advantage of newly available techniques in liquid chromatography-mass spectrometry (LC-MS) to analyze the proteomes of mammalian cells in response to ELF- and RF-EMF exposures. With technological advances in LC-MS and computational methods to analyze the resulting data, it has become possible to identify and to quantify thousands of proteins in a single shotgun proteomics experiment. Semi-quantitative proteomics with metabolic labeling of proteins such as the stable isotope labeling with amino acids in cell culture (SILAC) is a firmly established and accurate method to interrogate the complex and dynamic nature of proteomes [22]. In a typical SILAC experiment, tens of thousands of peptides and thousands of (non-redundant) proteins are reliably identified and quantified from mass spectrometry data, for example, using the widely-used MaxQuant/Andromeda software [23, 24]. We present the proteome-wide analyses of human fibroblasts and osteosarcoma cells, and of mouse embryonic stem cells exposed to ELF- and RF-EMFs. Our study indicates that if there is an effect of these EMF exposures, it is smaller than technical variation in a rigorously controlled triple-state SILAC approach. We find that less than 1% of the quantitated human or mouse proteome is differentially regulated in response to these EMFs and discuss the possible biological significance of this subtle response.

## Materials and methods

### Cell lines and culture conditions

To prepare cell lysates for triple-state (triplex) SILAC LC-MS analysis, human osteosarcomas (U2OS), human fibroblasts (VH10) and mouse embryonic stem cells (IB10) were grown in medium containing stable isotope-labeled arginine and lysine. Growth medium consisted of arginine- and lysine-free DMEM (PAA laboratories), supplemented with 10% dialyzed fetal bovine serum (FBS), 50 units/ml penicillin, 50 μg/ml streptomycin and 2 mM ultraglutamine (all from Gibco, Life Technologies), 1x non-essential amino acids (Lonza), 200 μM labeled arginine and 400 μM labeled lysine. The isotope combinations used for labeling were [^12^C_6_,^14^N_2_]-lysine (Lys-0) and [^12^C_6_,^14^N_4_]-arginine (Arg-0) for “Light” labeling; [4,4,5,5 D_4_]-lysine (Lys-4) and [^13^C_6_]-arginine (Arg-6) for “Medium” labeling; and [^13^C_6_,^15^N_2_]-lysine (Lys-8) and [^13^C_6_,^15^N_4_]-arginine (Arg-10) for “Heavy” labeling Cambridge Isotope Laboratories). In addition, for IB10 cells the medium was supplemented with 1000 U/ml leukemia inhibitory factor and 100 μM β-mercaptoethanol, and the cells were grown on dishes coated with 0.1% gelatin. Cells were grown for at least five generations at 37°C and in a humidified environment containing 5% CO_2_, and either directly used for treatment, or stored in liquid N_2_.

To prepare samples obtained from independent ELF exposures for immunoblot analysis, VH10 cells were grown in DMEM supplemented with 10% FBS, 50 units/ml penicillin, and 50 μg/ml streptomycin (Gibco, Life Technologies). The cells were grown to 40% confluency on 9 cm dishes before exposure. As control cell line for MutLα expression, HEK293T-Lα cells [25] were grown in the same medium as the VH10 cells, supplemented with 100 μg/ml Zeocin (Invivogen) and 300 μg/ml Hygromycin B (Roche). To turn off the expression of MutLα, HEK293T-Lα cells were exposed to 50 ng/ml doxycycline (Sigma) for one week.

### Highly-controlled *in vitro* EMF exposures

Labeled mammalian cells were subjected to low frequency (ELF) and radio frequency (RF: WiFi and UMTS) electromagnetic fields with sham-exposed cultures as controls. For the ELF exposure experiments, cells were incubated for 15 hrs in two shielded coil systems (chambers) of the IT’IS sXcELF apparatus [26, 27]. The apparatus was placed inside a Heracell 240I incubator (Thermo Scientific), which ensured constant environmental conditions (37°C, 5% CO_2_, 95% humidity) by two fans in both Mu-metal chambers. Temperature differences between the ELF exposed and sham-exposed cells were kept below 0.1°C. In one of the chambers cells were exposed to intermittent ELF signal (cycles of 5 min ON and 10 min OFF using 50 Hz power-transmission line signal including electrical pollution due to high frequency components from transients up to 1 kHz, with B = 2 mT RMS) with a non-uniformity of 1% (SD) for all possible culture dish locations. The other chamber was used for sham exposures, with fields <-43 dB (<0.05%) compared to ELF exposure. The exposure was carried out in a blinded fashion; the output files including the coil assignment from the sXcELF apparatus were decoded by IT’IS after LC-MS analysis. During the exposure, the current in the coils and the temperature in the chambers were continuously monitored.

For the RF exposure experiments, cells were incubated simultaneously in three identical exposure chambers placed next to each other within a well-insulated climate-controlled room. The exposure chambers consisted of large, stainless steel cabinets with inner dimensions 2.3 × 1.2 × 1.1 m^3^ (height × width × depth) in which incubator units were fitted in the upper part of the cabinets, including connections for climate control to the outside of the cabinets. Constant environmental conditions (37°C, 5% CO_2_, 95% humidity) were ensured by cycling humidified air through the incubators. In two exposure chambers cells were exposed to UMTS (2.1 GHz, E = 45 V/m RMS) and WiFi (5.8 GHz, E = 9.5 V/m RMS) signals for 24 hrs. It is noted that the field strength for 5.8 GHz is a factor of five lower mainly because of lower RF-power in combination with higher propagation and cable losses at 5.8 GHz. The field strength values were obtained by measurements at the location of the cells without Petri dishes. The antennae were placed in the bottom part of the chambers, such that the distance between them and the cells in the incubators was approximately 1.5 meters. This means that for the largest wavelength (14cm) the cells are at least 10 wavelengths from the antenna panel. Considering the antenna structure this set-up is expected to provide far-field conditions at the exposed cells. In the third chamber cells remained unexposed. Further details of the stainless steel cabinets, control of the antennae and the applied electromagnetic field homogeneity measurements for the cabinets will be described elsewhere. The antenna used for UMTS was FPA19-55V/448 manufactured by European Antennas. The antenna for 5.8 GHz WiFi, FPA16-2350V/1232, was also obtained from European Antennas. Signal generators (SMBV100) were from Rohde & Schwarz. The UMTS amplifier (10 W), ZH-2122H was obtained from RF-links, as was the AMP-7000/X 5.8 GHz WiFi amplifier (5 W). Cells were processed for LC-MS analysis immediately following (sham) exposure to the ELF- and RF-EMFs.

### Sample preparation and LC-MS analysis

After EMF exposure, cells were washed twice with ice-cold PBS, harvested and lysed in 250 μl lysis buffer containing 50 mM ammonium bicarbonate, 7 M urea, 2 M thiourea (Sigma Aldrich) and Complete^™^ protease inhibitor cocktail (Roche) at the recommended concentration in ultra-pure water (Baxter Healthcare). DNA was sheared by passing the lysate 20 times through a 25G needle. The lysate was cleared by centrifuging for 10 minutes at 14,000 *g* in an Eppendorf centrifuge. Total protein concentrations were determined using the NI^™^ protein assay kit (G-Biosciences). Equal amounts of protein (150 μg) of the differentially labeled lysates were mixed for SILAC-based LC-MS analysis consisting of two independent experiments: i) unexposed “Light” (denoted as L0) and “Medium” (denoted as M0) lysates were mixed with exposed “Heavy” lysates (denoted as H1), and ii) exposed “Light” (denoted as L1) and “Medium” (denoted as M1) lysates were mixed with unexposed “Heavy” lysate (denoted as H1). The resulting protein lysates were reduced, alkylated and digested with trypsin (Promega, sequencing grade) as described previously [28]. Peptides were fractionated by hydrophilic interaction liquid chromatography and the fractions were collected for mass spectrometry analysis.

Peptides were analyzed on an EASY-nLC system coupled with a Q Exactive^™^ mass spectrometer (Thermo Scientific). Peptide mixtures were trapped on a ReproSil C18 reversed phase column (Dr Maisch GmbH; column dimensions 2 cm × 100 μm, packed in-house) at a flow rate of 8 μl/min. Peptide separation was performed on ReproSil C18 reversed phase column (Dr Maisch GmbH; column dimensions 15 cm × 75 μm, packed in-house) using a linear gradient from 0 to 50% B (A = 0.1% formic acid; B = 80% (v/v) acetonitrile, 0.1% formic acid) in 180 min at a constant flow rate of 300 nl/min. The column eluent was directly electrosprayed into the mass spectrometer. Mass spectra were acquired in continuum mode; fragmentation of the peptides was performed in data-dependent acquisition mode by higher-energy collisional dissociation using the top 15 selection. Additional settings for the mass spectrometer operation were as follows: MS resolution at 70,000; MS AGC target 3E6; MS maximum injection time of 100 ms; MS scan range of 375–1,400 m/z; MS/MS resolution at 17,500; MS/MS AGC target 1E5; MS/MS maximum injection time of 200 ms; and intensity threshold 5E3.

### Protein identification and quantitation

Thermo Xcalibur RAW files (423 GB in total) were analyzed using the MaxQuant software (version 1.3.0.5) integrated with the Andromeda peptide search engine [23, 24]. Peptide fragmentation spectra were searched with trypsin specificity, with legitimate cleavages before proline (Trypsin/P), against the corresponding species-specific library of protein sequences (89796 human proteins and 53213 mouse proteins from the UniProtKB database release 2015_02 [29]) including reverse sequences and common contaminants in the FASTA format (http://maxquant.org/contaminants.zip, 247 proteins). Peptides were at least 7 amino acids long and were allowed to contain up to two missed cleavages and five modifications per tryptic peptide. Oxidation of methionine and N-terminal acetylation were set as variable modifications, and carbamidomethylation of cysteine was set as fixed modification. The precursor and fragment ion mass tolerances were set at 6 ppm and at 20 ppm, respectively. Peptide and protein identifications were obtained with 1% false discovery rate (FDR) thresholds. Two or more protein accessions were grouped together whenever the sets of identified peptides of the proteins were either identical or inclusive (subsets). Protein quantifications required at least two razor and/or unique peptides, including those with the variable modifications.

### Data management and statistical analyses

First, MaxQuant result files, such as the peptide (evidence.txt) and protein lists (proteinGroups.txt), and the searched human/mouse protein sequence library (*.fasta) were uploaded to and integrated by the PIQMIe proteomics server [30] (http://piqmie.biotools.nl). Specifically, this web server transformed each EMF exposure data set into a relational database (SQLite version 3; http://sqlite.org) to facilitate efficient data access and downstream analyses such as the detection of differentially regulated proteins and of potentially perturbed cellular processes (pathways) upon EMF exposures.

Furthermore, we implemented an array of established statistical methods for differential expression analysis as command-line tools in R and Python languages. The analysis relied on SILAC protein ratios corrected for unequal protein loading (referred to as normalized ratios) and fully quantitated protein groups associated with six normalized ratios from reciprocal SILAC experiments: i) four 'treated' ratios from exposed *versus* unexposed (sham) samples, and ii) two 'control' ratios from exposed *versus* exposed samples and sham *versus* sham samples. The significance of protein fold-changes was assessed using three outlier detection approaches namely the peak intensity-based significance B [23], standard Z-score and its robust version called M-score [31, 32]. In addition, we used two rank-based (non-parametric) methods implemented in the fcros (version 1.2) [33] and RankProd (version 2.42.0) [34, 35] R packages as well as one linear modeling approach with empirical Bayes estimation implemented in the limma R package (version 3.26.0) [36, 37]. A series of fold-change (FC) and/or *p*-value thresholds was applied to each method to detect differentially regulated proteins at increasing levels of stringency (FC > 1.2 or 1.5; (two-tailed) *p*-value < 0.1, 0.05 or 0.01, which corresponds to |Z| > 1.65, 1.96 or 2.58). The *p*-values were adjusted by the Benjamini–Hochberg method [38] to control the FDR of the analysis.

Importantly, our experimental design based on triplex SILAC with two label-swaps was explicitly taken into account in the differential protein expression analysis by “encoding” this design into a contrast matrix as used in the limma method or using a composite Boolean filter based on fold-changes in the other methods. Both approaches ensured that only proteins with consistent changes in the 'treated' SILAC ratios but with relatively smaller or no changes in the 'control' ratios were detected as potential candidates. Furthermore, median absolute deviation (MAD) was used as a robust measure of variability in both 'treated' and 'control' SILAC protein ratios, and also served as proxy to estimate the signal-to-noise (S/N) ratio of the detection (approximated by MAD_treated_ *versus* MAD_control_ ratio). The greater the S/N ratio the higher the reliability of detecting differentially regulated proteins.

Finally, differentially regulated proteins detected in the mammalian proteomes in response to EMF exposures were subjected to an improved model-based protein set analysis using the mgsa R package (version 1.18.0) [39] in order to infer potentially perturbed biological processes (as defined by the UniProt Gene Ontology Annotation database (UniProt-GOA) release 142 for human and release 128 for mouse, March 2015 [40] and cellular pathways (as defined by two curated pathway databases: the KEGG PATHWAY release 73.0+/03-10, March 2015 [41]; and the Reactome version 52, March 2015 [42]). Specifically, the mgsa analysis was performed separately for mouse and human proteins detected as regulated in response to EMFs: i) ELF, UMTS or WiFi, ii) ELF and RFs (combined results from UMTS and WiFi exposures) and iii) all EMFs (combined results from ELF, UMTS and WiFi exposures). Note that the latter two instances approximate the scenarios in which mammalian cells would be exposed to more than one type of EMF simultaneously; however, these in vitro EMF exposures were not conducted in our study. In the resulting Bayesian networks, the marginal posterior probabilities were estimated using a Markov chain Monte Carlo method with 20 independent runs of the sampler, each with 10^6^ iterations. A cellular process or pathway was inferred as perturbed only if its marginal posterior probability was greater than 0.5. Note that a higher posterior indicates stronger support in the Bayesian framework, which is in contrary to the standard hypothesis-based approaches (in which a lower *p*-value indicates a higher level of confidence).

### Antibodies and quantitative immunoblotting

Cell lysates containing a total amount of 5 μg protein were mixed with SDS sample buffer (2% SDS, 10% glycerol, 60 mM Tris-HCl, pH 6.8), resolved on an 8% polyacrylamide Tris-Glycine gel and transferred to an immobilon-P PVDF membrane (Millipore). The membrane was blocked in 3% skim milk powder (Fluka) in PBS/0.1% Tween20 and cut into sections according to the molecular weight marker to be able to probe for individual proteins. Blocked membranes were incubated over night at 4°C with primary antibody, washed in PBS/0.1% Tween20 and incubated with horseradish peroxidase-conjugated secondary antibody for two hours. Primary antibodies were mouse-anti-MLH1 (PharMingen, 550838) diluted 1:1000, rabbit-anti-PMS2 (Abcam, EPR3947) diluted 1:1000, and mouse-anti-α tubulin (Sigma, T5168) diluted 1:2500. Secondary antibodies were donkey-anti-rabbit (Jackson immunoresearch, 711-035-152) diluted 1:2500, and sheep-anti-mouse (Jackson Immunoresearch, 515-035-003) diluted 1:2500. After washing with PBS/0.1% Tween20, the membrane was incubated with ECL substrate (GE Healthcare) and imaged with the Alliance imaging system (Uvitec Cambridge). Intensities of observed protein bands were quantified using the Fiji open-source platform for biological-image analysis [43]. Within each sample, expression levels of DNA mismatch repair protein MLH1 were normalized against the α-tubulin loading control. To compare the MLH1 levels between samples, the expression levels were normalized against the average value of unexposed (sham) samples (L0, M0 and H0).

### Data and software availability

All mass spectrometry-based proteomics data were deposited in the ProteomeXchange Consortium [44] through the PRIDE partner repository [45] with the database identifier PXD002862. The post-processed proteomics data were also made freely available for queries through the PIQMIe server (http://piqmie.biotools.nl/results/*<dataset>*, where *dataset* refers to one of the six EMF exposures: ELF_human, ELF_mouse, UMTS_human, UMTS_mouse, WIFI_human or WIFI_mouse). The TIFF images taken from the immunoblots and the intensities of the observed protein bands in these blots are provided in supplementary materials (S1 File). The source codes of the bioinformatics tools (in R and Python languages) developed to analyze semi-quantitative proteomics data, such as those from *in vitro* exposures to EMFs, can be found on GitHub and/or CERN's Zenodo platform (PIQMIe version 1.0, http://dx.doi.org/10.5281/zenodo.34090; EMF-DM version 1.0.1, http://dx.doi.org/10.5281/zenodo.166705).

## Results

### Experimental design

**Fig 1:**
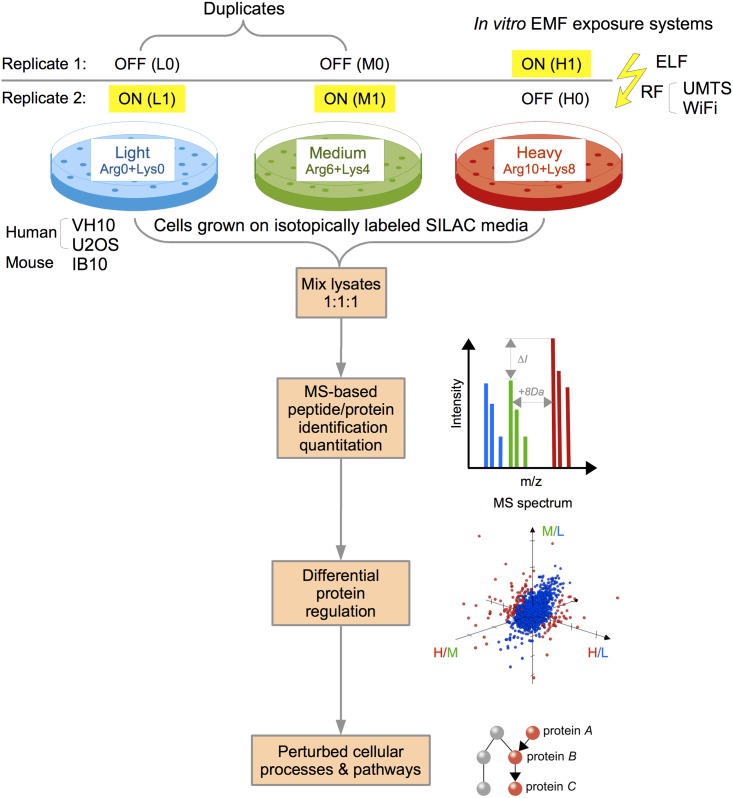
Experimental setup to study proteome-wide biological responses to non-ionizing EMFs using semi-quantitative mass spectrometry. Triple-state (triplex) SILAC proteomics with reverse metabolic labeling of human fibroblasts (VH10), human osteosarcomas (U2OS) and mouse embryonic stem cells (IB10) exposed to different EMFs with extremely low (ELF) or radio frequencies (UMTS or WiFi). Cells were cultured in media containing “Light” (Arg-0/Lys-0), “Medium” (Arg-6/Lys-4) and “Heavy” (Arg-10/Lys-8) stable isotopes. Cultures were sham (denoted as L0, M0 and H0) or exposed (denoted as L1, M1 and H1) to EMFs. Two independent LC-MS experiments of mixtures of cell extracts were performed: two sham and one exposed extract in the mixture (L0+M0+H1, indicated as replicate 1), and two exposed extracts with one sham extract in the mixture (L1+M1+H0, indicated as replicate 2). Note that the L and M samples (duplicates) were treated equally in both experiments and could therefore be used as internal controls to quantify the experimental variation due to cell culturing, metabolic labeling and/or preparing the samples for mass spectrometry analysis. Further downstream bioinformatics analyses involved peptide/protein identification and quantitation, and the detection of differentially regulated proteins and perturbed cellular processes or pathways.

In order to detect cellular responses upon exposures to non-ionizing EMFs, we implemented an unbiased and highly sensitive mass spectrometry-based proteomics approach. Our semi-quantitative proteomics experiments involved triple-state SILAC with reverse (swap) metabolic labeling of three mammalian cell lines, each exposed to three different non-ionizing EMFs (Fig 1).

The choice of mammalian cell lines reflects different biological signatures that are relevant in response to EMF exposures: the VH10 human fibroblasts, a skin cell line used as a model for the first tissue that would be exposed to the fields, in particular to EMFs in radio frequency range; the U2OS osteosarcoma cell line as a sensitive cell line with compromised molecular regulatory pathways in cancer; and the mouse IB10 embryonic stem cells as sensitive sensors for subtle disturbances during cellular differentiation, as these cells have the potential to become any cell type in the adult organism.

We implemented a triple-state SILAC approach with reverse labeling to be able to detect putative small effects on protein abundance induced by EMF exposures. Changes in protein abundance between the “Heavy” samples on the one hand and the “Light” and “Medium” samples on the other hand were statistically evaluated when the changes were consistently observed in the same direction in both replicates (L0+M0+H1 and L1+M1+H0).

### Proteomics data analysis workflow

**Fig 2:**
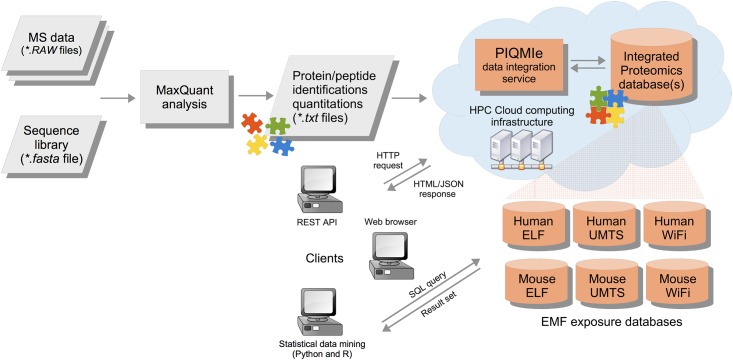
Semi-quantitative proteomics data management and analysis. The SILAC-based mass spectrometry data from EMF exposed mammalian cells were analyzed by the MaxQuant/Andromeda software. The resulting peptide/protein identifications and quantifications were uploaded to the PIQMIe proteomics server, which integrated the EMF exposure data with protein information from UniProtKB and made the databases available for user-driven queries and statistical analyses.

We developed the PIQMIe proteomics server for reliable management, statistical analysis and visualization of semi-quantitative mass spectrometry data (Fig 2). In particular, our SILAC-based EMF exposure data on three mammalian cell lines were analyzed and made available for queries through web-based graphical and programmatic user interfaces. Importantly, the PIQMIe web service automates common post-processing tasks, which are often performed manually by researchers such as summarizing peptide/protein identifications and quantifications, filtering out decoy hits and known contaminants, and/or log-transforming SILAC protein ratios. Moreover, users can perform specific queries on the resulting database(s), for example, retrieving proteins newly identified in a proteomics experiment which have previously not been verified experimentally at the protein level according to the evidence in the UniProtKB database. Furthermore, we implemented an array of established statistical methods to infer differential protein regulation and perturbed cellular pathways in the mammalian cells due to EMF exposure. These methods were directly coupled with the resulting databases to aid in reproducible analyses of the EMF exposure data.

### Summarizing the SILAC-based EMF exposure experiments

The mass spectrometry data obtained from our SILAC experiments were summarized in terms of identified and quantitated peptides/proteins using the PIQMIe proteomics server [30]. About one-fourth of the human or mouse proteome (as defined by the UniProtKB database) was identified by the MaxQuant/Andromeda analysis (Table 1, S1–S3 Tables), with mean coverage of 27.30% for human and of 25.35% for mouse cells. Approximately half of the identified protein accessions belong to the high-quality and manually curated UniprotKB/Swiss-Prot entries (53% for mouse *versus* 48% for human).

**Table 1:** Summary of mass spectrometry-based protein identifications in human and mouse proteomes exposed to EMFs.

Proteome	Database/section	Number of protein accessions	ELF	UMTS	WiFi
			Number of protein ident.(%)	Number of protein ident. (%)	Number of protein ident.(%)
Human	UniProtKB/Swiss-Prot	42077	12558 (29.85)	10708 (25.45)	11434 (27.17)
	UniProtKB/TrEMBL	47719	13861 (29.05)	12249 (25.67)	12740 (26.70)
	**UniProtKB**	**89796**	**26419 (29.42)**	**22957 (25.57)**	**24174 (26.92)**
Mouse	UniProtKB/Swiss-Prot	24724	6919 (27.98)	7518 (30.41)	7044 (28.49)
	UniProtKB/TrEMBL	28489	6132 (21.52)	6588 (23.12)	6265 (21.99)
	**UniProtKB**	**53213**	**13051 (24.53)**	**14106 (26.51)**	**13309 (25.01)**

Note that the splice isoforms are included in the protein counts but decoy hits and contaminants are excluded from the counts; the UniProtKB/Swiss-Prot section contains highly curated entries (accessions) whereas the UniProtKB/TrEMBL section is unreviewed.

Further comparisons of the protein sets identified in different EMF exposures showed high overlap and/or similarity, as measured by the Jaccard index (*J*) between the sets (Table 2 and S1 Fig). In particular, the RF protein sets (UMTS and WiFi) shared higher similarity with each other than did either RF set with the ELF set, which was in accordance with the experimental procedures used.

**Table 2:** Two- and three-way set comparisons of protein identifications from the EMF exposure experiments.

**Human**							
**A**	**B**	**|A|**	**|B|**	**|A** ∩ **B|**	**|A** ∪ **B|**	**% Overlap**	***J***
						100 * |A ∩ **B| / MAX(|A|, |B|)**	**|A** ∩ **B| / |A** ∪ **B|**
ELF	UMTS	26419	22957	20711	28665	78.39	0.72
ELF	WiFi	26419	24174	21503	29090	81.39	0.74
UMTS	WiFi	22957	24174	20702	26429	85.64	0.78
**Mouse**							
ELF	UMTS	13051	14106	11365	15792	80.57	0.72
ELF	WiFi	13051	13309	11108	15252	83.46	0.73
UMTS	WiFi	14106	13309	12154	15261	86.16	0.80
**Human**							
**A**	**B**	**C**	-	**|A** ∩ **B** ∩ **C|**	**|A** ∪ **B** ∪ **C|**	**% Overlap**	***J***
						100 * |A ∩ B ∩ C| / MAX(|A|, |B|, |C|)	**|A ∩ B ∩ C| / |A ∪ B ∪ C|**
ELF	UMTS	WiFi	-	19389	30023	73.39	0.65
**Mouse**							
ELF	UMTS	WiFi		10570	16409	74.93	0.64

Note that the Jaccard index (*J*) indicates the similarity between the sets (values between 0 and 1, i.e. complete dissimilarity and identity, respectively); the overlap score (%) indicates the percentage of protein identifications common to the sets with regard to the largest one; set operations such as union (∪), intersection (∩) and cardinality (|…|) are indicated in the headers.

The protein identifications including their isoforms were further clustered into (non-redundant) protein groups by the MaxQuant/Andromeda software, resulting in up to 4933 and 5286 protein groups in mouse and human cells, respectively (Table 3). For human samples the most protein groups were detected in the ELF data set, whereas the least protein groups were detected in the UMTS data set; and *vice versa* for mouse samples. In general, the majority of protein groups (54–86%) was associated with at least one SILAC protein ratio based on two or more peptide quantitation events. However, there were more proteins quantitated in mouse than in human cells when exposed to the same EMF, mainly due to more tryptic peptides identified/quantitated in the mouse samples. Of the two human cell lines, the U2OS cells had generally more quantitated peptides (up to 29% more) and proteins (up to 18% more) than the VH10 cells had.

**Table 3:** Summary of (non-redundant) protein identifications/quantifications in human (VH10 and U2OS) and mouse (IB10) cells exposed to EMFs.

EMF	Cell line	Number of protein ident.	Number of protein quant. (%)	Number of decoys	Number of contaminants
ELF	U2OS	5286	^**a**^3237 (61.24)	97	125
			^**b**^3288 (62.20)		
			^**c**^2927 (55.37)		
	VH10	5286	^**a**^3114 (58.91)	97	125
			^**b**^3305 (62.52)		
			^**c**^2854 (53.99)		
	IB10	4583	^**a**^3276 (71.48)	71	96
			^**b**^3389 (73.95)		
			^**c**^3055 (66.66)		
UMTS	U2OS	4551	^**a**^3722 (81.78)	76	134
			^**b**^3656 (80.33)		
			^**c**^3416 (75.06)		
	VH10	4551	^a^3233 (71.04)	76	134
			^b^3023 (66.42)		
			^c^2761 (60.67)		
	IB10	4933	^a^4199 (85.12)	66	113
			^b^4035 (81.80)		
			^c^3812 (77.28)		
WiFi	U2OS	4841	^a^3656 (75.52)	71	130
			^b^3803 (78.56)		
			^c^3483 (71.95)		
	VH10	4841	^a^3294 (68.04)	71	130
			^b^3336 (68.91)		
			^c^3028 (62.55)		
	IB10	4662	^a^4010 (86.01)	62	112
			^b^3958 (84.90)		
			^c^3704 (79.45)		

Note that the splice isoforms are included in the protein counts but decoy hits and contaminants are excluded from the counts; the superscripts indicate protein quantifications with at least one SILAC ratio in replicate 1 (L0+M0+H1)^a^, replicate 2 (L1+M1+H0)^b^ or with complete SILAC quantifications in both replicates^c^ (six protein ratios in total).

Interestingly, about one-third of the identified mouse proteins—an order of magnitude more than in the human proteome—have previously not been verified experimentally at protein level according to the evidence in the UniProtKB database (S4 Table). This result indicates that the MS-based protein identification of the mouse proteome has not yet reached the point of saturation compared to the human proteome.

### Dissecting the effects of EMF exposures on mammalian cells

**Fig 3:**
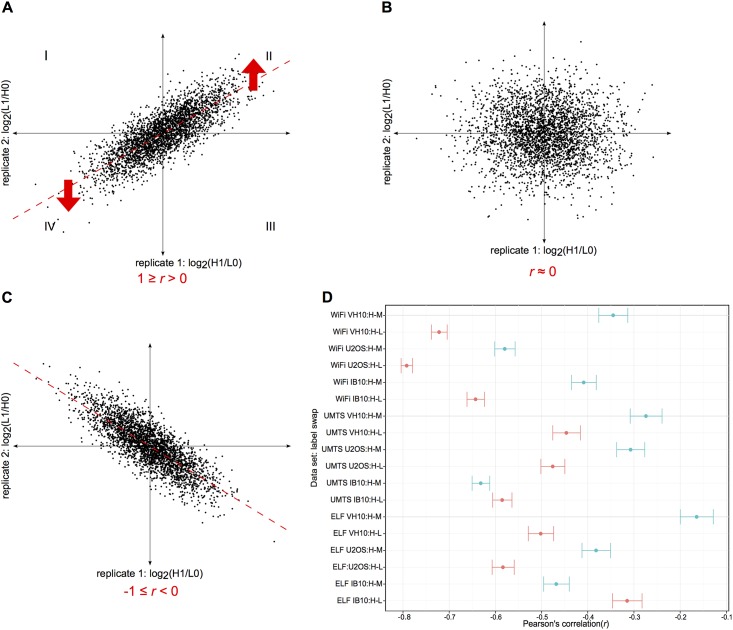
Correlation analysis of reciprocal SILAC protein quantifications in three mammalian cell lines exposed to three different EMFs. (A-C) Three scatterplots based on simulated SILAC protein ratios from reverse labeling experiments—with treated (H1 or L1) *versus* sham (L0 or H0) samples on both axes—illustrate three possible scenarios in which the scatter depends on the effect of a treatment (e.g. EMF exposure) *versus* SILAC reverse labeling: (A) the scatter is in the direction of the treatment, as indicated by a positive Pearson's correlation coefficient (*r*); (B) the scatter does not have an identifiable trend, as indicated by a value of *r* close to zero; (C) the scatter is in the direction of the SILAC reverse labeling, as indicated by a negative value of *r*. Each scatterplot is divided into four quadrants (I-IV): proteins with inconsistent SILAC reciprocal ratios are located in the I and III quadrants whereas proteins with consistent up- and down-regulation upon treatment are located in the II and IV quadrants, respectively. (D) The dot plot summarizes quantitative data from human (U2OS and VH10) and mouse (IB10) cell lines exposed to ELF, UMTS and WiFi (the individual scatterplots are shown in S2–S4 Figs); the *r* estimates including the error bars (95% confidence interval) are based on SILAC protein ratios from the H-M (in blue) and the H-L (in red) reverse labeling experiments. Note that the estimated *r* values are negative in all EMF exposures and hence correspond to the third scenario illustrated by the scatterplot (C).

Our experimental design enabled to distinguish the variability in SILAC protein ratios introduced by the EMF exposure from that introduced by the SILAC swap labeling procedure and therefore to infer subtle perturbations in the exposed mammalian proteomes. The overall correlation analysis of reciprocal SILAC protein ratios (exposed *versus* sham) from the replicate experiments consistently resulted in a pattern of inversely associated protein ratios (Fig 3), as indicated by the negative Pearson's correlation coefficients (*r*) for all the EMF data sets (*r* between -0.79 and -0.16, 95% CI, *p*-value < 0.001). Importantly, this analysis followed by visualization using scatterplots (S2–S4 Figs) suggested that the EMF exposures had overall a smaller effect on the quantitated mammalian proteomes than the SILAC swap labeling procedure. However, the correlation analysis did not rule out the possibility of detecting differential protein regulation in response to the EMF exposures. Seven out of nine H-L swap experiments shared stronger (inverse) associations than the H-M swaps (as indicated by a larger absolute |*r|*, with mean difference of 0.17, 95% CI of 0.04 to 0.30, t(8) = 2.96 and *p*-value < 0.05 according to the paired t-test), indicating a label-specific bias and the importance of using double-swap rather than single-swap SILAC labeling.

**Fig 4:**
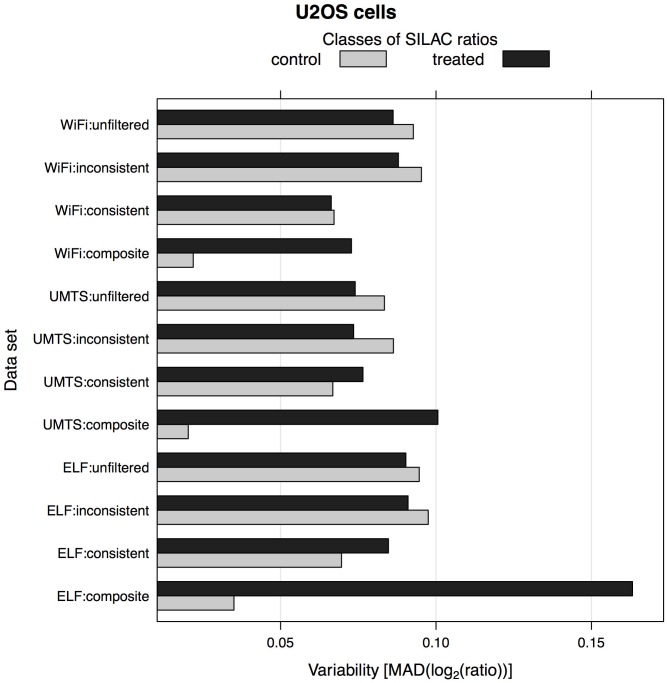
Variability of SILAC protein ratios in human U2OS cells upon ELF, UMTS and WiFi exposures. Median absolute deviation (MAD) is used as a robust measure of variability in SILAC protein ratios. Note that *unfiltered* refers to an unfiltered SILAC data set containing all quantitated protein groups; *(in)consistent* refers to a filtered SILAC data set of protein groups with (in)consistent ratios in both reverse labeling experiments, this fold-change filtering procedure is only possible because of the duplex SILAC design; *composite* refers to a filtered SILAC data set of protein groups with greater 'treated' ratios than 'control' ratios (in total there are four 'treated' ratios from exposed *versus* sham samples, and two 'control' ratios from exposed *versus* exposed samples and sham *versus* sham samples), this (composite) fold-change filtering procedure requires triplex SILAC design. The results for the human VH10 and mouse IB10 cells are shown in S5 Fig.

Next, we assessed the feasibility of detecting differential protein expression by comparing the variability of SILAC protein ratios for treated *versus* control conditions, and by estimating the signal-to-noise ratio (S/N) in the filtered *versus* unfiltered triplex SILAC data. This analysis showed that by applying the composite fold change-based filter on protein quantifications the S/N ratio (refer to Materials and Methods) increased significantly across all EMF exposure data sets, thereby enabling improved detection of potentially regulated proteins upon the EMFs (Fig 4 and S5 Fig). For example, the S/N ratio of the partially filtered SILAC data (denoted as WiFi:consistent) of human U2OS cells remained relatively low compared to that of the unfiltered data set (denoted as WiFi:unfiltered). However, the S/N ratio improved significantly for the fully filtered data set (denoted as WiFi:composite) because of the triplex SILAC design. Therefore, our triplex SILAC experiments with two label-swaps (H-L and H-M) enabled more reliable downstream analyses of the EMF exposure data than would a simpler, duplex SILAC design with one label-swap.

### Differential protein regulation and perturbed cellular pathways

Instead of relying on results obtained by a single method, we used an array of established statistical methods to infer, as reliably and sensitively as possible, differentially regulated proteins in the mammalian proteomes in response to non-ionizing EMFs (Table 4). This ensemble-based approach resulted in the detection of 45 differentially regulated proteins, of which 18/14/13 proteins were potentially affected by the ELF/UMTS/WiFi exposure, respectively. However, most of these proteins were associated with changes smaller than 1.5-fold (*p*-value < 0.1). Having SILAC data on three distinct mammalian cell lines *per* EMF exposure enabled to assess the biological reproducibility and/or consistency of differential protein regulation across multiple cell lines of the same and/or different species.

**Table 4:** Differentially regulated proteins detected upon EMFs in human (VH10 and U2OS) and mouse (IB10) cells using an array of statistical methods.

EMF	Cell line	FC	FC+sigB	Z-score	M-score	RankProd	fcros	limma	Combined (union) set	Number of proteins
ELF	VH10	^2^MLH1, ^1^WDR75	^2^MLH1	MLH1	**MLH1**, UBE2A, WDR75	^2^**MLH1**, ^2^LEPREL2, ^1^NUMB, ^1^WDR75, ^1^GINS1	^2^MLH1	-	↑: MLH1, UBE2A, WDR75, ^a^LEPREL2, ^a^NUMB, ^a^GINS1	6
	U2OS	^1^AMPH, ^1^DNMT1	-	-	METAP2,DNMT1,NT5C2	^1^**DHX33**, ^1^**AMPH**, ^1^**TBL3**, ^1^**MVK**, ^1^**DNMT1**, ^1^LIMD1	^2^AMPH, ^1^DHX33, ^1^MVK, ^1^DNMT1	-	↑: MVK, TBL3, ^b^DHX33	8
									↓: AMPH, DNMT1, METAP2, NT5C2, ^b^LIMD1	
	IB10	^1^Rhot1	-	-	Glmn, Rhot1	^2^Glmn, ^1^Cryzl1, ^1^Ap1m1	-	-	↑: Glmn, Rhot1, Cryzl1, Ap1m1	4
UMTS	VH10	^1^SPAG7	-	-	SPAG7	^2^**EXOC2**, ^2^**CRYZL1**, ^1^**SPAG7**, ^1^DPY30	^2^EXOC2, ^1^KDM1A, ^1^SPAG7	-	↑: SPAG7	5
									↓: EXOC2, DPY30, KDM1A, ^a^CRYZL1	
	U2OS	^1^TWISTNB, ^1^MBOAT7	^1^TWISTNB, ^1^MBOAT7	-	**MBOAT7**, EXOC2, MOGS, PKP2	^2^**TWISTNB**, ^1^MBOAT7, ^1^H2AFY	^2^TWISTNB, ^1^MBOAT7	-	↓: MBOAT7, EXOC2, MOGS, H2AFY, ^b^PKP2, ^b^TWISTNB	6
	IB10	^1^Calcoco1, ^1^Pcf11, ^1^Acbd6	-	-	Acbd6, Calcoco1	^2^**Acbd6**, ^1^**Calcoco1**, ^1^Pcf11, ^1^Wipi2	^2^Acbd6, ^1^Calcoco1, ^1^Pcf11	-	↑: Calcoco1, Pcf11	4
									↓: Acbd6, Wipi2	
WiFi	VH10	^1^LEO1, ^1^PNPO	-	-	-	^1^**LEO1**, ^1^**PNPO**, ^1^ANKRD28, ^1^KRAS	^1^LEO1, ^1^PNPO	^1^PNPO	↑: ANKRD28	4
									↓: LEO1, ^a^PNPO, ^a^KRAS	
	U2OS	^1^AKAP8L	-	-	AKAP8L	^2^**AKAP8L**, ^1^**NUCKS1**	^2^AKAP8L, ^1^NUCKS1	-	↓: AKAP8L, ^b^NUCKS1	2
	IB10	^2^Drosha, ^1^Zfp57, ^1^Atxn7l3b	^2^Drosha	Drosha	**Atxn7l3b**, **Drosha**, Zfp57	^2^**Drosha**, ^1^**Atxn7l3b**, ^1^**Zfp57**, ^1^**Nde1**, ^1^Wwc2, ^1^Asf1a, ^1^Scaf8	^2^Drosha, ^1^Zfp57, ^1^Atxn7l3b, ^1^Nde1	-	↑: Drosha, Atxn7I3b, Wwc2, Nde1, Asf1a	7
									↓: Zfp57, Scaf8	

Three classes of statistical methods used: i) outlier detection using protein fold-changes (FC) assessed with peak intensity-based significance B (sigB), Z-score and M-score; ii) rank-based (non-parametric) RankProd and fcros; and iii) an improved linear modeling approach with empirical Bayes estimation, limma/TREAT. A non-statistical FC-based approach (without *p*-value estimation) was also included. (↑) Up- and (↓) down-regulated proteins detected with varying degrees of stringency: FC > ^1^1.2 or ^2^1.5, and/or (adjusted) *p*-value < 0.1, 0.05 or **0.01**

^a,b^ indicate a protein with incomplete (or no) SILAC quantifications in human ^a^U2OS or ^b^VH10 cells upon the same EMF exposure. Note that only the leading proteins of the non-redundant groups are listed here and referred by their official gene symbols (http://www.genenames.org). Further details about the proteins such as UniProtKB accession numbers and SILAC ratios are presented in S5 Table.

According to this protein-centric analysis, the majority of the differentially regulated proteins were identified in a single cell line, except the human exocyst complex component 2 (EXOC2) and the quinone oxidoreductase-like protein 1 (CRYZL1) including its mouse ortholog. While the down-regulation of EXOC2 upon UMTS exposure was consistent in both VH10 and U2OS human cells, the regulation of CRYZL1 was inconsistent; the human CRYZL1 protein was down-regulated upon UMTS exposure whereas its mouse ortholog was up-regulated upon ELF exposure. Furthermore, we ranked the statistical methods according to the numbers of regulated proteins detected: RankProd returned the largest set (38 proteins), followed by fcros (21 proteins), M-score (19 proteins), fold-change assessed with peak intensity-based significance B (4 proteins), Z-score (2 proteins) and limma/TREAT (1 protein). About one-third of the differentially regulated proteins were confirmed by two distinct classes of—mostly outlier detection and rank-based—methods while the remaining majority of the proteins were singletons. Among the proteins inferred by the majority of statistical methods were the human DNA mismatch repair protein MLH1, lysophospholipid acetyltransferase 7 (MBOAT7) and the mouse ribonuclease 3 (DROSHA).

**Fig 5:**
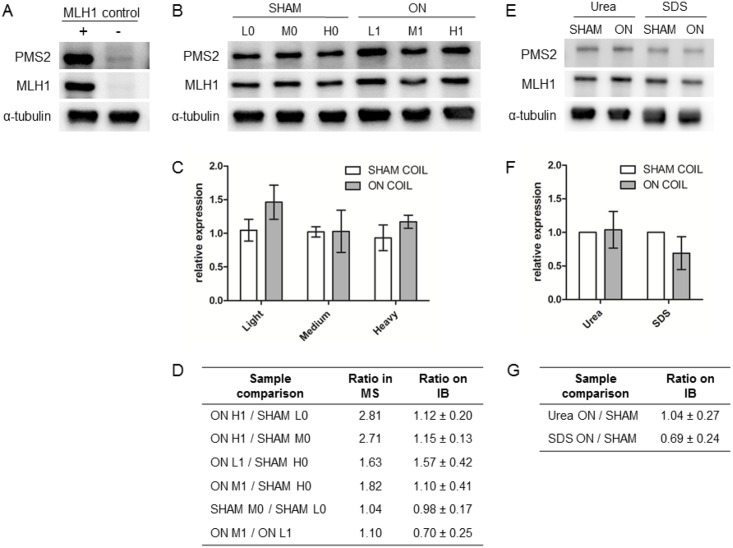
Quantitative immunoblot analysis of MLH1 expression in the VH10 cell line upon ELF exposure. (A) Immunoblot of control cell line HEK293T-Lα [25] in which expression of DNA mismatch repair protein MLH1 and its binding partner PMS2 is regulated by doxycycline. The presence (+) and absence (-) of doxycycline is indicated. (B) Immunoblot of extracts from untreated (SHAM) and exposed (ON) VH10 cells labeled with different isotopes (L, M and H) and used for semi-quantitative mass-spectrometry analysis. Proteins were visualized using antibodies against MLH1, its binding partner PMS2, and α-tubulin as loading control. (C) Relative MLH1 expression levels (mean ± SD) in extracts from untreated and exposed cells as determined from 6 immunoblot replicas of the MS samples. (D) Tabulated ratios for relative MLH1 expression levels in differentially labeled VH10 cells as determined by mass spectrometry (MS) and immunoblot (IB) analyses. (E) Immunoblot of VH10 cells, lysed using either urea or SDS, obtained from an independent exposure. (F) Relative MLH1 expression levels (mean ± SD) in extracts from an independent exposure as determined from 9 immunoblot replicas. (G) Tabulated ratios for relative MLH1 expression levels based on immunoblot analysis of extracts from the independent exposure.

We selected the protein MLH1 from the set of all differentially regulated proteins (Table 4) as the best candidate to validate the results from the LC-MS analysis for the following reasons: i) it was detected by nearly all statistical methods (except limma) as being up-regulated in VH10 human fibroblasts upon ELF exposure, ii) had the largest and most significant fold-change and iii) was identified as an integral part of the Gene Ontology term association network that we describe below. Specifically, MLH1 is involved in DNA mismatch repair, which is an important process required for maintaining genome stability. Loss of DNA mismatch repair in humans predisposes to Lynch syndrome with a high incidence of colonic and endometrial cancers [46]. MLH1 forms a stable heterodimeric MutLα complex with the PMS2 protein; however, PMS2 becomes unstable in the absence of MLH1 [46] (Fig 5A). First, we analyzed the amount of MLH1 and PMS2 in the cell extracts which were differentially labeled with Light/Medium/Heavy amino acids and were used for the LC-MS analysis (Fig 5B and 5C). This revealed that in these samples the increase in the amount of MLH1 upon ELF exposure based on quantitative immunoblot analysis (0.98–1.57-fold) is lower than indicated by the mass spectrometry-based analysis (1.04–2.81-fold), and is not statistically significant (Fig 5D) according to the two-way ANOVA analysis (α = 0.05, *p*-values > 0.05). This result was confirmed by quantitative immunoblot analysis of MLH1 levels in VH10 cells which were exposed to ELF in an independent experiment (Fig 5E–5G). Thus, the DNA mismatch repair protein MLH1 is not up-regulated upon ELF exposure.

**Fig 6:**
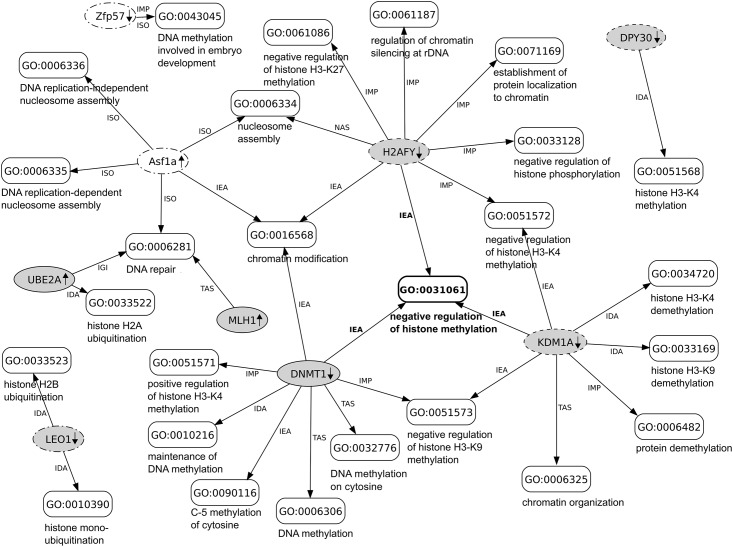
Network of differentially regulated mammalian proteins associated (annotated) with biological processes. In this network, the proteins and biological processes (referred by the Gene Ontology (GO) terms) are indicated by oval- and box-shaped nodes, respectively; human proteins (gray) and mouse (white) proteins detected as differentially regulated upon ELF (solid), UMTS (dashed) and WiFi (dot-dashed) exposures; protein-to-GO term associations are indicated by edges labeled with GO evidence codes: inferred from direct assay (IDA); inferred from mutant phenotype (IMP); inferred from genetic interaction (IGI); traceable author statement (TAS); non-traceable author statement (NAS); inferred from sequence orthology (ISO); inferred from electronic annotation (IEA). For additional details on the proteins refer to S5 Table.

In addition to the protein-centric analysis described above, we also investigated in which biological processes or related pathways are the differentially regulated proteins involved (Table 4). To infer potentially perturbed cellular pathways in the exposed mammalian cells, we performed an improved model-based protein set analysis [39] coupled with three complementary and curated databases namely UniProt-GOA, KEGG PATHWAY and Reactome. Using the UniProt-GOA database, this analysis inferred only one significantly perturbed biological process—referred by the Gene Ontology term 'negative regulation of histone methylation' (GO:0031061; marginal posterior probability greater than 0.5; 0.65 ± 0.02 SD)–in human but not mouse cells, and only in the scenario, in which the cells would be exposed to all three EMFs simultaneously (for the details refer to “Data management and statistical analyses” in the methods section). Specifically, three down-regulated proteins (with fold-changes between 1.2 and 1.5 and adjusted *p*-values < 0.1) namely the human H2A histone family member Y (H2AFY; upon UMTS exposure), lysine-specific histone demethylase 1A (KDM1A; upon UMTS exposure) and DNA (cytosine-5-)methyltransferase 1 (DNMT1; upon ELF exposure) were found associated with negative regulation of histone methylation. This 'core' set of proteins was expanded with other differentially regulated proteins from both species (Table 4) and visualized in a network of associated biological processes, including those processes with marginal posterior probabilities below 0.5. In this network, most of the biological processes were related to chromatin modification and DNA repair (Fig 6) and the majority of protein-to-Gene Ontology term associations (20 out of 35) were annotated with experimental evidence from literature. However, unlike the UniProt-GOA, the mgsa analysis did not infer any significant pathways according to the KEGG and Reactome databases.

## Discussion

In this study we present the first proteome-wide semi-quantitative mass spectrometry analysis of human and mouse cells exposed to three different non-ionizing EMFs, namely ELF, UMTS and WiFi, using highly-controlled and standardized *in vitro* exposure systems. Given the high numbers of identified and quantitated proteins in the mammalian proteomes, our SILAC-based experiments were successful and in good agreement with published proteomics studies [47, 48]. We implemented a triplex SILAC design with two label-swaps (H-L and H-M) that enabled more reliable protein- and pathway-level analyses of potential cellular perturbations upon EMF exposures compared to simpler experimental designs (such as duplex SILAC with a single label-swap). Importantly, the triplex SILAC design was taken explicitly into account in the differential expression analysis, resulting in improved signal-to-noise ratio of the detection, which ensured that only proteins with consistent changes in the 'treated' protein ratios and with relatively smaller or no changes in the 'control' protein ratios were selected as putative candidates.

The correlation analysis of protein quantifications across all reciprocal SILAC experiments consistently resulted in a pattern of inversely associated protein ratios (Fig 3) and thus suggested that the effect of the EMFs on the mammalian proteomes is smaller than that of the SILAC swap labeling. As this analysis did not rule out the possibility of detecting differentially regulated proteins upon EMF exposures, we performed an extensive differential protein expression analysis using an array of established statistical methods. This ensemble-based approach suggested that less than 1% of the quantitated human and mouse proteomes respond to the EMF exposures by small changes in protein abundance (mostly by less than 1.5-fold). This indicates that the EMFs have a subtle bearing on the mammalian cells.

Although most of the statistical methods used have primarily been developed to assess differential gene expression in one- or two-color microarrays, they have been recently benchmarked on and successfully accommodated to semi-quantitative proteomics data, in particular those obtained from experiments with few replicates [49–51]. Our study also underlines the importance of using more than one method for differential protein expression analysis. The numbers of significant protein hits returned by the individual methods were rather low. Moreover, same proteins inferred by multiple methods were found associated with different *p*-values. By combining the results of different statistical methods we gained sensitivity and therefore obtained a larger set of potentially regulated proteins than using a single method alone. Note that it was not our aim to systematically benchmark these methods nor to select a single “best” method in terms of sensitivity and specificity but to detect proteins whose abundance is most likely regulated by exposure to EMFs.

One of the most prominent candidates based on comprehensive statistical analyses of our SILAC-based mass spectrometry data, the human DNA mismatch repair protein MLH1, was selected for the follow-up experimental validation using immunoblot analysis. The immunoblot analyses of the same samples as used for the semi-quantitative mass spectrometry experiments, however, did not reveal a significant change in the level of this protein upon ELF exposure (Fig 5). Furthermore, concomitant up-regulation of its obligatory partner protein PMS2 was not detected in the immunoblot analysis. Consistent with these results are the following observations that i) the change in the abundance of MLH1 was detected exclusively in VH10 human fibroblasts based on our mass spectrometry data analysis, and ii) an *in vitro* study [52] that assessed the putative effect of ELF exposure on DNA replication and transcription, did not detect an effect on the efficiency of DNA mismatch repair. Importantly, this outcome exemplifies that the possible identification of false positive candidates has to be taken into account even when setting up high-throughput studies as rigorously controlled as triple-state SILAC with two label-swaps, and underscores the need for (targeted) experimental validation.

As we did not confirm the change in the abundance of the DNA repair protein MLH1 in the follow-up immunoblot experiments, we embarked on an computational approach that used all available mass spectrometry-based data from our cell lines and/or EMF exposures. Specifically, all differentially regulated proteins detected in the human and mouse cell lines were subjected to an improved model-based protein set analysis that, in comparison to single-term association approaches, takes the statistical dependence between ontological terms into account in a Bayesian network [39]. The results of this exploratory data analysis are presented in Fig 6. Interestingly, a single biological process that comes into focus is chromatin metabolism in general and chromatin modification events related to epigenetic control of gene expression in particular [53].

The analysis suggests that the histone variant macro-H2A.1, which is encoded by the *H2AFY* gene, is down-regulated upon UMTS exposure. This histone variant is associated with repressive chromatin and cellular senescence [54]. Interestingly, the histone chaperone ASF1A is involved in forming macro-H2A.1 containing chromatin [55]. This histone chaperone is identified as up-regulated in mouse cells upon WiFi exposure. Furthermore, DNA- and histone-modifying enzymes are implicated by the analysis, such as DNA (cytosine-5-)-methyltransferase 1 (DNMT1) and lysine-specific histone demethylase 1A (KDM1A, also known as LSD1). Deficiencies in the former enzyme have been associated with cancer and developmental disorders [56], as well as with DNA mismatch repair deficiency, among others, through reduced expression of the DNA repair protein MLH1 [57]. However, MLH1 expression is not reduced according to our semi-quantitative mass spectrometry and immunoblot analyses. The lysine-specific histone demethylase 1A can affect methylation status of histone H3 (namely H3K4 and H3K9), thereby affecting transcriptional status of genes [58]. Moreover, deficiency in this histone demethylase (KDM1A) is implicated in cancer and embryonic stem cell differentiation. An interesting link between the DNA- and histone-modifying enzymes is the fact that lysine-specific histone demethylase 1A (KDM1A)-mediated demethylation of a lysine on DNA (cytosine-5-)-methyltransferase 1 (DNMT1) stabilizes the latter enzyme [59].

In addition to the lysine-specific histone demethylase 1A, which affects the methylation status of histone H3K4, the analysis also pointed to potential down-regulation of the histone methyltransferase complex regulatory subunit (DPY30) in VH10 human fibroblasts upon UMTS exposure. This protein is part of different methyltransferase-containing complexes, including the MLL1/MLL complex, which contains the catalytic subunit MLL1 (also known as KMT2A) that acts on histone H3K4 [60, 61]. Furthermore, the RNA polymerase-associated protein (LEO1) is detected as down-regulated in human fibroblasts upon WiFi exposure. As part of the PAF1/RNA polymerase II complex, LEO1 is implicated in the regulation of development and maintenance of embryonic stem cell pluripotency [62]. In particular, PAF1 complex stimulates transcription of the *KMT2A/MLL1* gene and recruits the ubiquitin-conjugating enzyme 2A (UBE2A) to chromatin [63], a post-translational modification enzyme, which is detected as differentially (up-)regulated in human fibroblasts upon ELF exposure.

Given the inability to biochemically verify a possible EMF-mediated increase in the levels of the DNA repair protein MLH1, which represents our strongest candidate emerging from the semi-quantitative mass spectrometry analysis the significance, if any, of an effect of EMF exposure on epigenetically controlled gene expression, as presented in Fig 6, remains unclear at the present time. However, if transcriptional programs could be affected by EMFs, then it could be expected that biological effects of EMF exposure would be very pleiotropic.

## Supporting information

S1 Fig Venn diagrams of human (A) and mouse (B) protein sets identified in different EMF exposures. (TIFF)

S2 Fig Scatterplots of reciprocal SILAC protein ratios from label-swap ELF-EMF exposure experiments. Number of protein groups (n); Pearson's correlation coefficient (*r*).
(TIFF)

S3 Fig Scatterplots of reciprocal SILAC protein ratios from label-swap UMTS-EMF exposure experiments. Number of protein groups (n); Pearson's correlation coefficient (*r*).
(TIFF)

S4 Fig Scatterplots of reciprocal SILAC protein ratios from label-swap WiFi-EMF exposure experiments. Number of protein groups (n); Pearson's correlation coefficient (*r*).
(TIFF)

S5 Fig Variability of SILAC protein quantifications in human VH10 and mouse IB10 cell lines exposed to EMFs. Median absolute deviation (MAD) is used as a robust measure of variability in SILAC protein ratios. Note that *unfiltered* refers to an unfiltered SILAC data set containing all quantitated protein groups; *(in)consistent* refers to a filtered SILAC data set of protein groups with (in)consistent ratios in both reverse labeling experiments, this fold-change filtering procedure is only possible because of the duplex SILAC design; *composite* refers to a filtered SILAC data set of protein groups with greater 'treated' ratios than 'control' rations (in total there are four 'treated' ratios from exposed *versus* sham samples, and two 'control' ratios from exposed *versus* exposed samples and sham *versus* sham samples), this fold-change (composite) filtering procedure requires triplex SILAC design.
(TIFF)

S1 Table Summary of peptide identifications and quantifications. Human (U2OS and VH10) and mouse (IB10) cell lines; *exp_name*–MS experiment name (prefixed with cell line); *n_pep_ids*–number of redundant peptide identifications, filtered for decoys and contaminants; *n_pep_qts*–number of redundant peptide quantifications; *n_unq_pep_seq+mod_ids*–number of non-redundant peptide identifications unique by sequence and modifications; *n_unq_pep_seq+mod_qts*–number of non-redundant peptide quantifications unique by sequence and modifications; *n_unq_pep_seq_ids*–number of non-redundant peptide identifications unique by sequence; *n_unq_pep_seq_qts*–number of non-redundant peptide quantifications unique by sequence; *n_pep_ids_decoys*–number of redundant peptides detected as decoys (false positives); *n_pep_ids_conts*–number of redundant peptides detected as contaminants; *n_unq_pep_seq_decoys*–number of non-redundant peptide decoys unique by sequence; *n_unq_pep_seq_conts*–number of non-redundant peptide contaminants unique by sequence.
(TXT)

S2 Table Summary of protein identifications. *n_prot_acc*–number of protein accessions including isoforms in the source database; *n_prot_ids*–number of MS-based protein identifications including splice isoforms, filtered for decoys and contaminants; *n_prot_acc_evid_protein–*number of protein accessions with protein-level evidence; *n_prot_acc_evid_transcript*–number of protein accessions with transcript-level evidence; *n_prot_acc_evid_homology*–number of protein accessions with homology-based evidence; *n_prot_acc_evid_predicted*–number of proteins predicted *in silico*; *n_prot_acc_evid_uncertain*–number of protein accessions with uncertain evidence.
(TXT)

S3 Table Summary of (non-redundant) protein identifications and quantifications. Human (U2OS and VH10) and mouse (IB10) cell lines; *exp_name*–MS experiment name (prefixed with cell line); *n_pgrp_ids*–number of non-redundant protein identifications, filtered for decoys and contaminants; *n_pgrp_qts*–number of non-redundant protein quantifications; *n_pgrp_ids_by_site*–number of non-redundant proteins identified by modification site; *n_pgrp_decoys*–number of non-redundant proteins detected as decoys (false positives); *n_pgrp_conts*–number of non-redundant proteins detected as contaminants.
(TXT)

S4 Table Summary of potentially new MS-based protein identifications. The numbers refer to human or mouse proteins with no previous experimental evidence at protein-level according to the UniProtKB database (release 2015_02). The numbers in parentheses indicate percentages (%) given all identified proteins *per* species and EMF exposure.
(TXT)

S5 Table Detailed information on the differentially regulated proteins detected upon EMF exposures. This table was obtained by querying the EMF databases via the PIQMIe service (refer to the 'Search Grid' tab). Note that the leading proteins (accessions) of non-singleton groups are in red; normalized SILAC protein ratios: H/L, H/M, M/L including their reciprocals; sequence coverage (*SeqCov*) expressed as the number of amino acids spanned by the quantitated peptides divided by the sequence length of the leading protein; the number of peptide quantifications (*Nq*), standard deviation of the mean log_2_-transformed peptide ratios (*SD*) and the peak intensity-base significance B (*SigB*, *p*-values adjusted by the Benjamini-Hochberg method).
(DOC)

S1 File TIFF images and protein intensities obtained from quantitative immunoblotting. (ZIP)
