# Supplementary figures and images for: Semi-quantitative proteomics of mammalian cells upon short-term exposure to non-ionizing electromagnetic fields
Source: PLoS One. 2017 Feb 24;12(2):e0170762. doi: 10.1371/journal.pone.0170762 (PMC5325209; doi:10.1371/journal.pone.0170762)

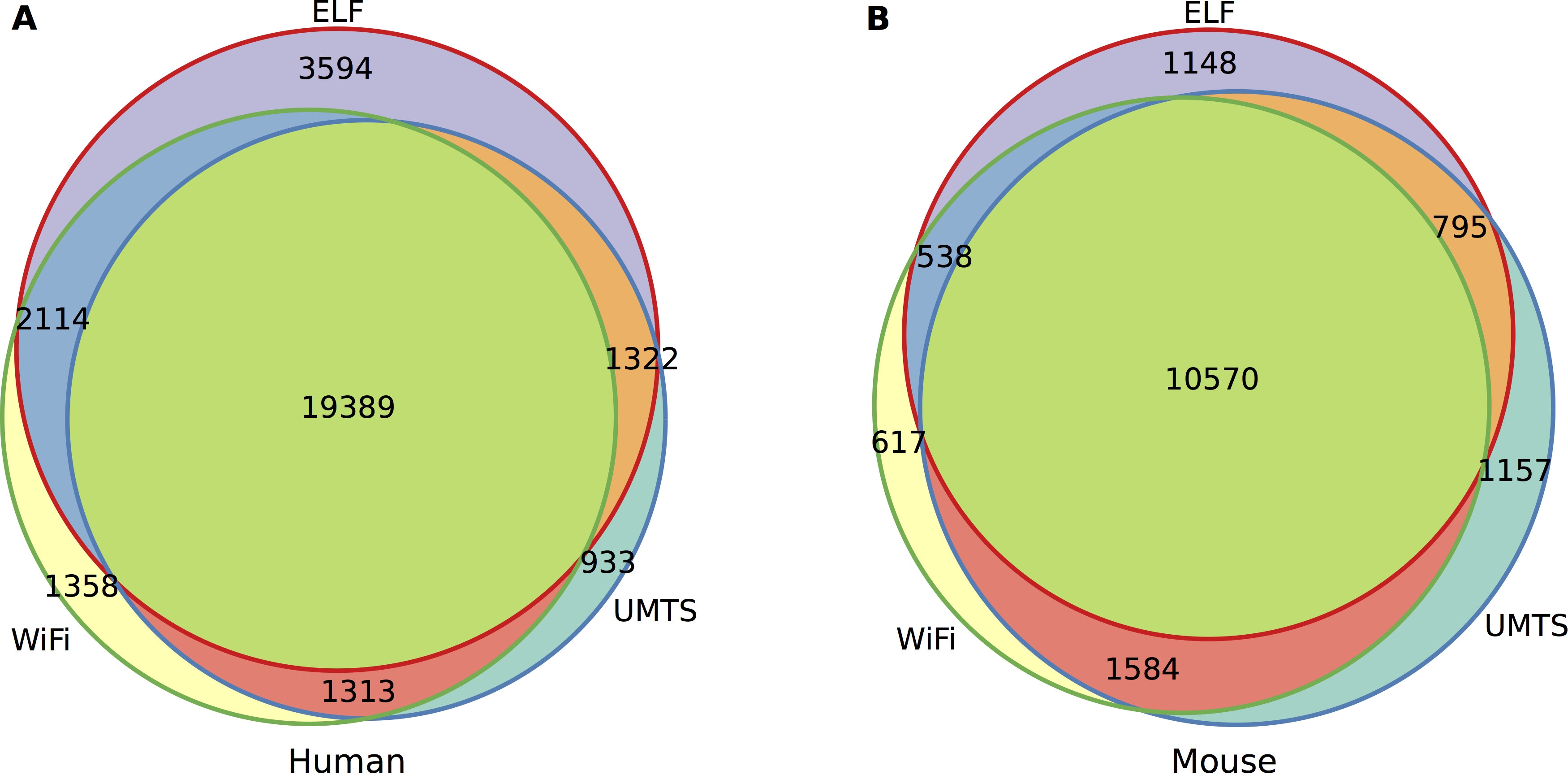

Supplement: S1 Fig — (TIFF) [file pone.0170762.s001.tiff]

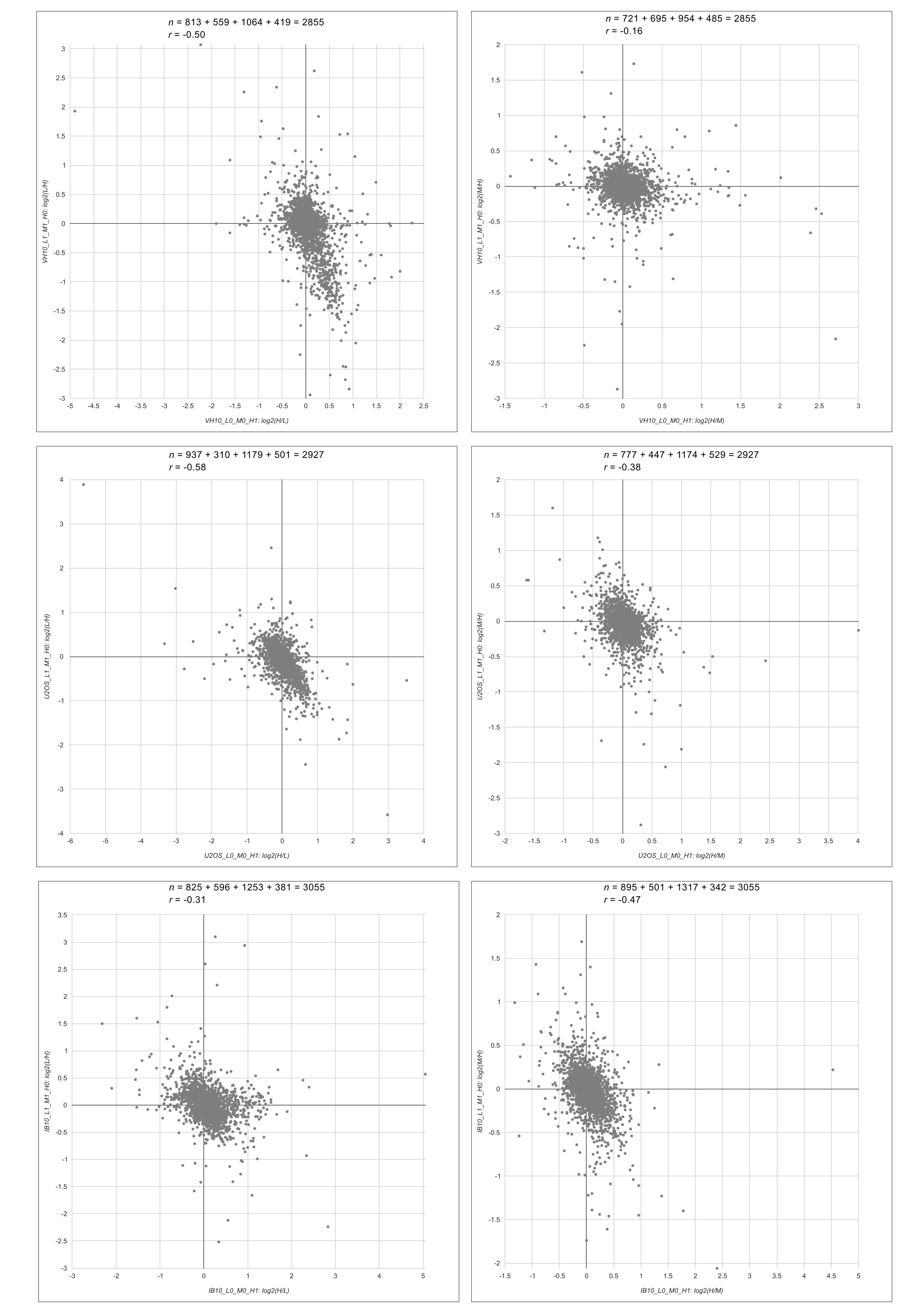

Supplement: S2 Fig — Number of protein groups (n); Pearson's correlation coefficient (r). (TIFF) [file pone.0170762.s002.tiff]

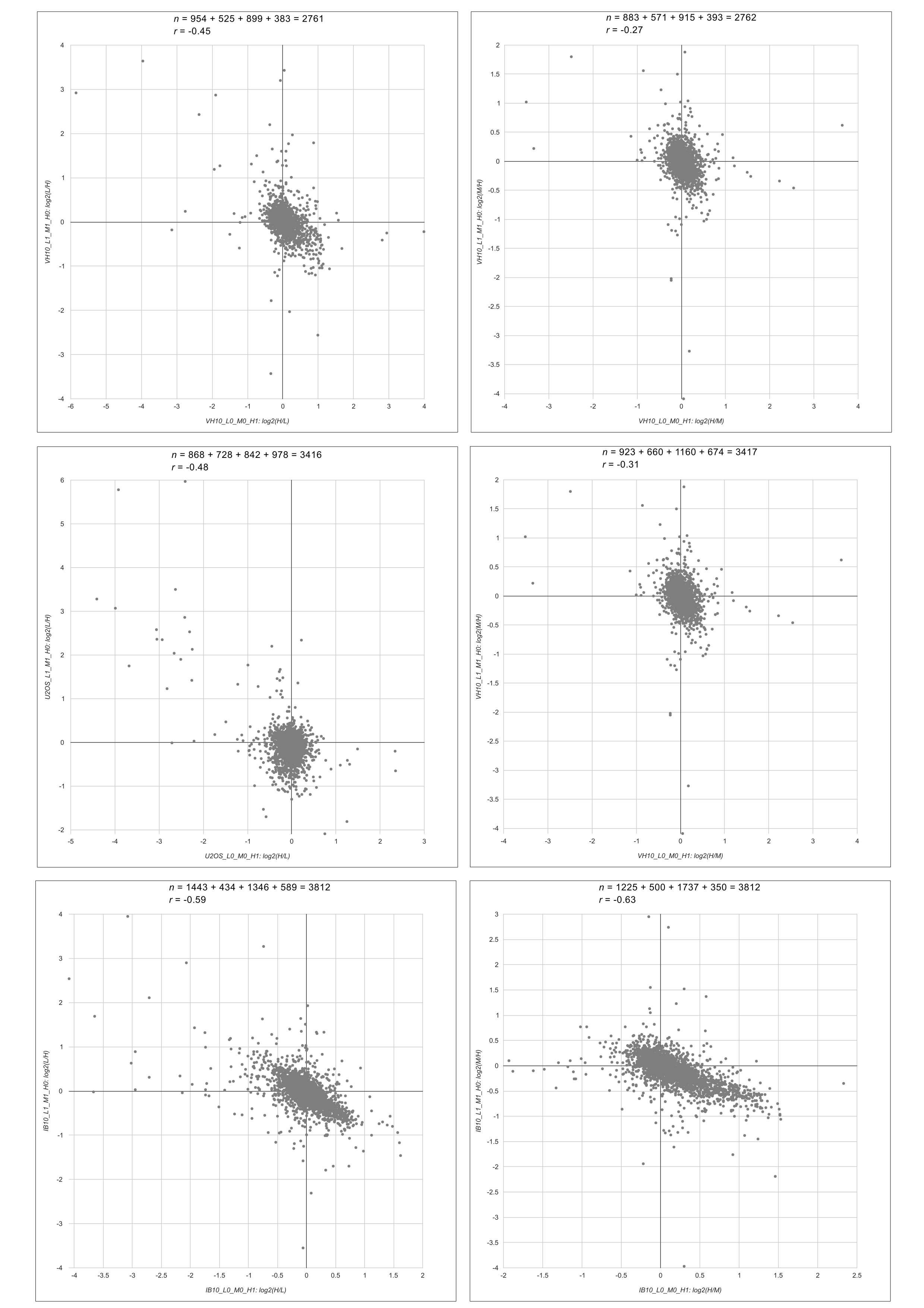

Supplement: S3 Fig — Number of protein groups (n); Pearson's correlation coefficient (r). (TIFF) [file pone.0170762.s003.tiff]

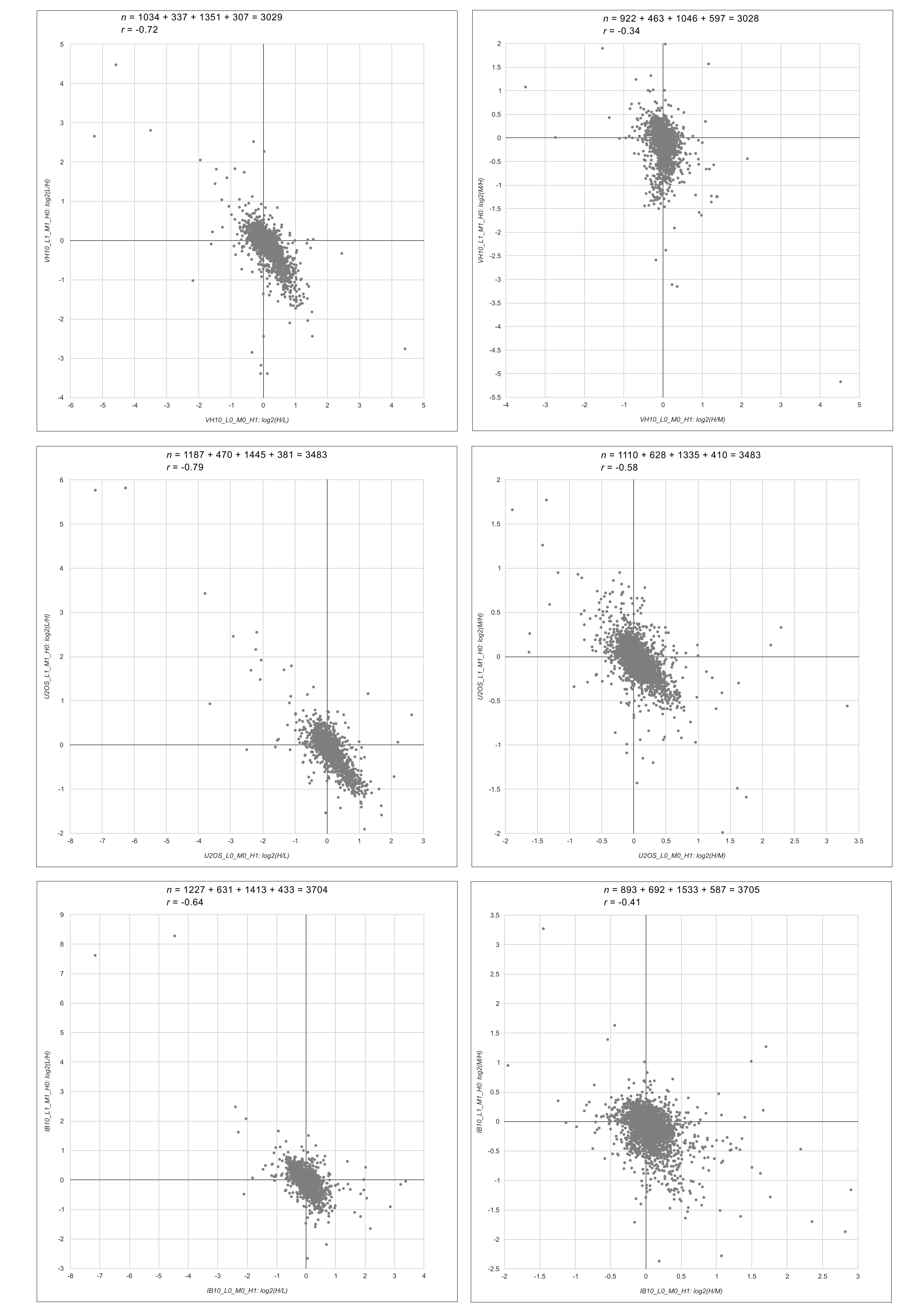

Supplement: S4 Fig — Number of protein groups (n); Pearson's correlation coefficient (r). (TIFF) [file pone.0170762.s004.tiff]

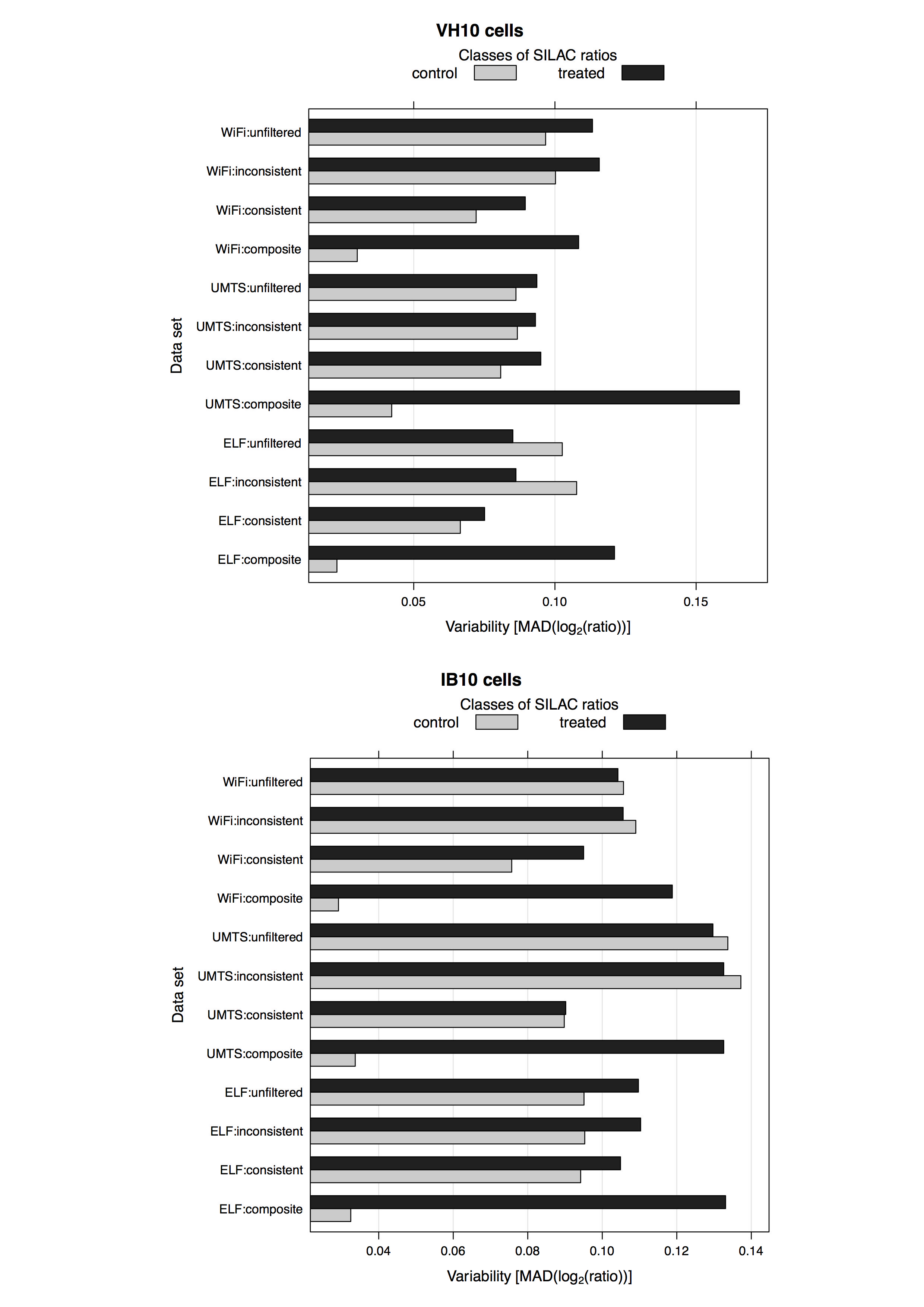

Supplement: S5 Fig — Median absolute deviation (MAD) is used as a robust measure of variability in SILAC protein ratios. Note that unfiltered refers to an unfiltered SILAC data set containing all quantitated protein groups; (in)consistent refers to a filtered SILAC data set of protein groups with (in)consistent ratios in both reverse labeling experiments, this fold-change filtering procedure is only possible because of the duplex SILAC design; composite refers to a filtered SILAC data set of protein groups with greater 'treated' ratios than 'control' rations (in total there are four 'treated' ratios from exposed versus sham samples, and two 'control' ratios from exposed versus exposed samples and sham versus sham samples), this fold-change (composite) filtering procedure requires triplex SILAC design. (TIFF) [file pone.0170762.s005.tiff]

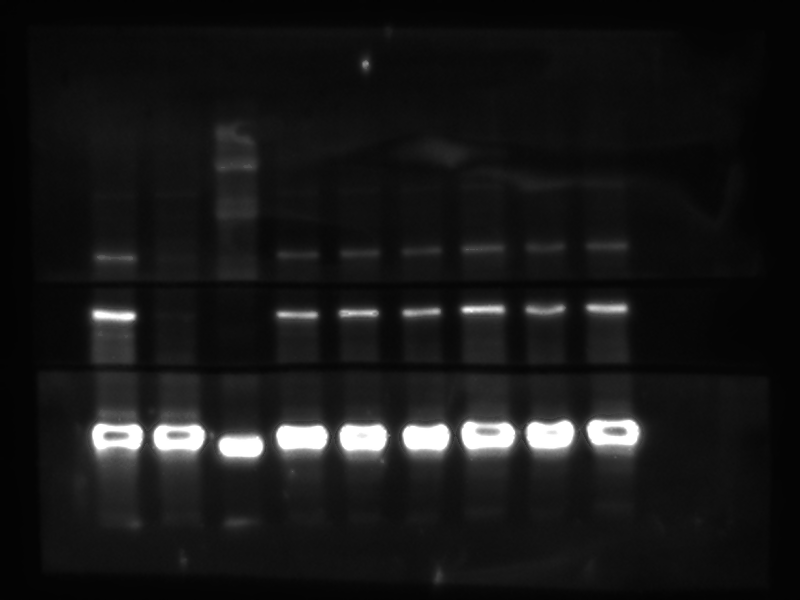

Supplement: S1 File — (ZIP) [file pone.0170762.s011.zip › immunoblot_analysis/images/030714_MSblot6_2.5min.tiff]

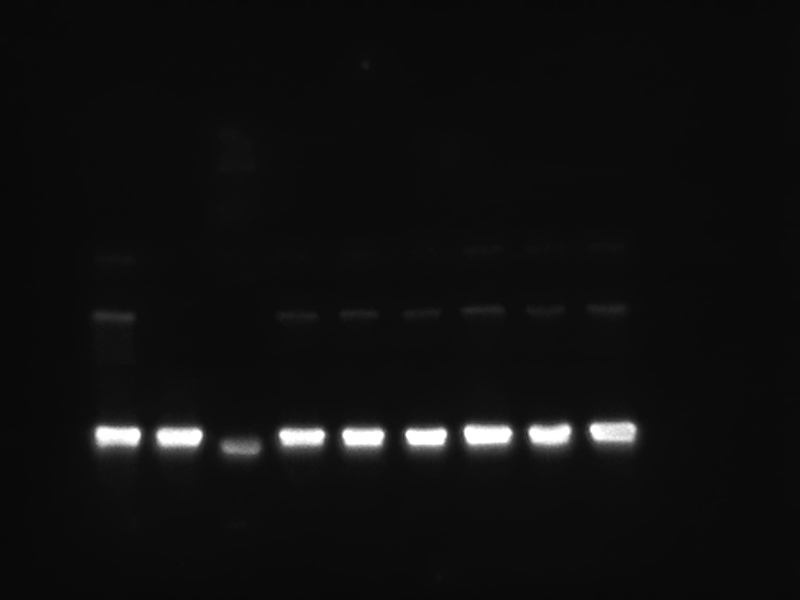

Supplement: S1 File — (ZIP) [file pone.0170762.s011.zip › immunoblot_analysis/images/030715_MSblot6_5sec.tiff]

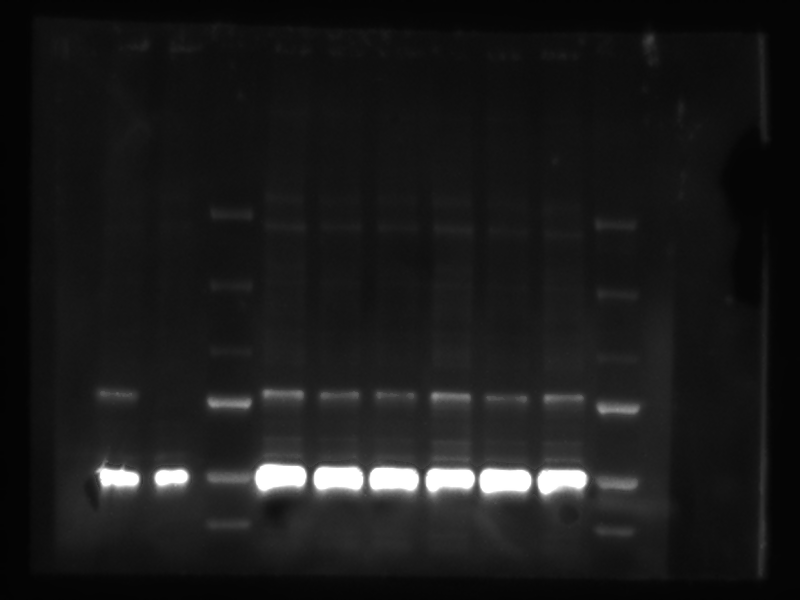

Supplement: S1 File — (ZIP) [file pone.0170762.s011.zip › immunoblot_analysis/images/120314_MSblot3_2min.tiff]

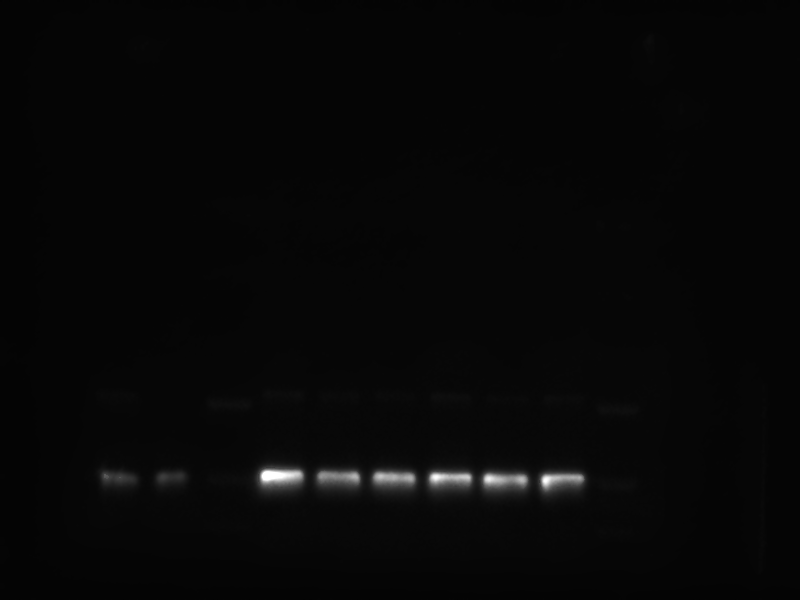

Supplement: S1 File — (ZIP) [file pone.0170762.s011.zip › immunoblot_analysis/images/120314_MSblot3_5sec.tiff]

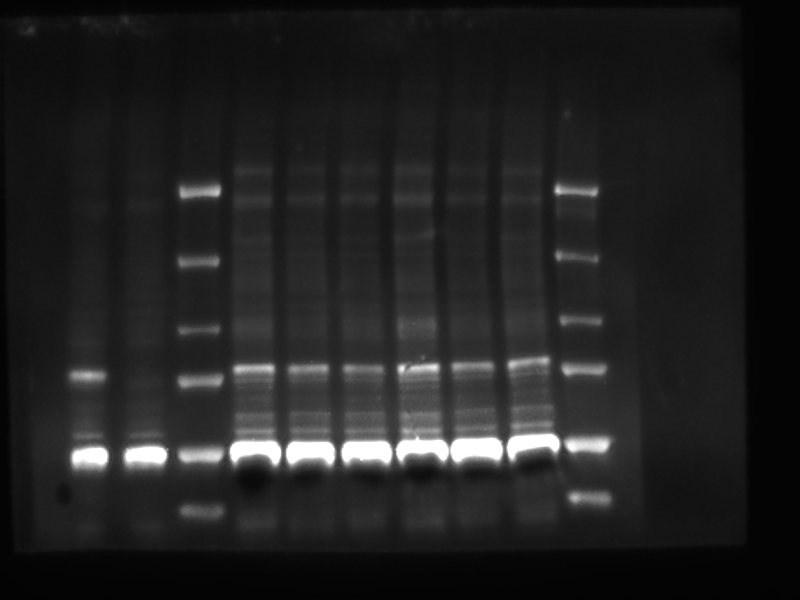

Supplement: S1 File — (ZIP) [file pone.0170762.s011.zip › immunoblot_analysis/images/120314_MSblot4_2min.tiff]

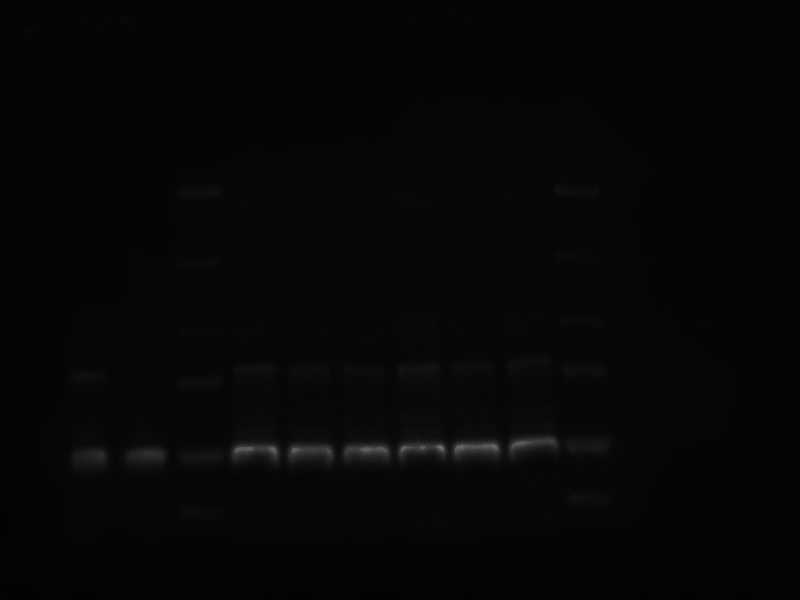

Supplement: S1 File — (ZIP) [file pone.0170762.s011.zip › immunoblot_analysis/images/120314_MSblot4_5sec.tiff]

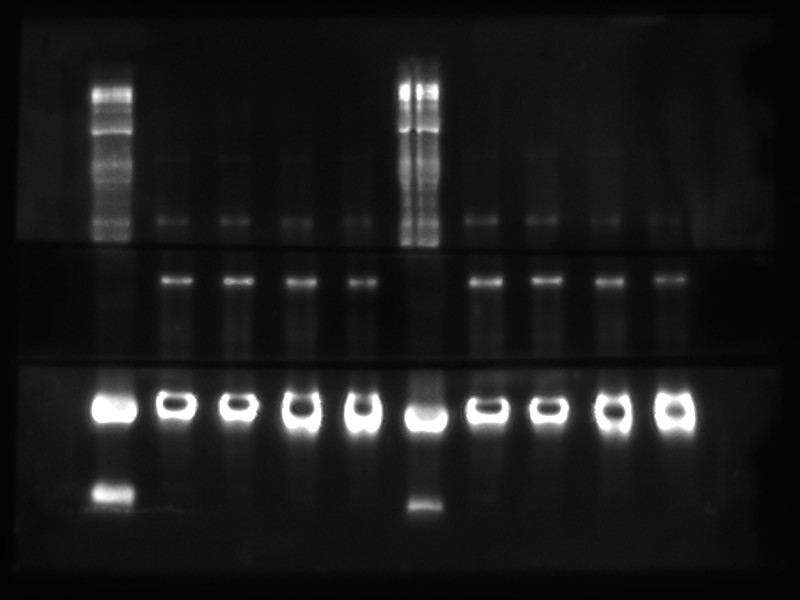

Supplement: S1 File — (ZIP) [file pone.0170762.s011.zip › immunoblot_analysis/images/170714_DuploBlot4_2.5min.tiff]

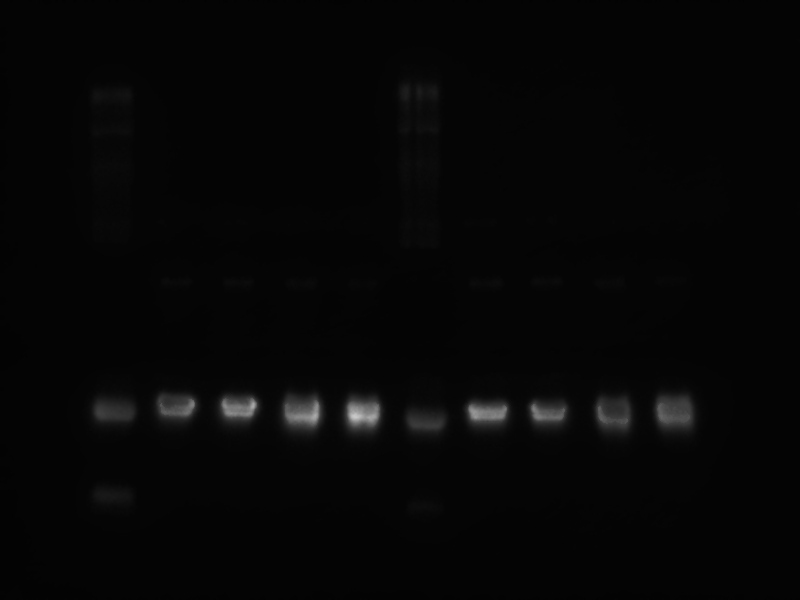

Supplement: S1 File — (ZIP) [file pone.0170762.s011.zip › immunoblot_analysis/images/170714_DuploBlot4_5sec.tiff]

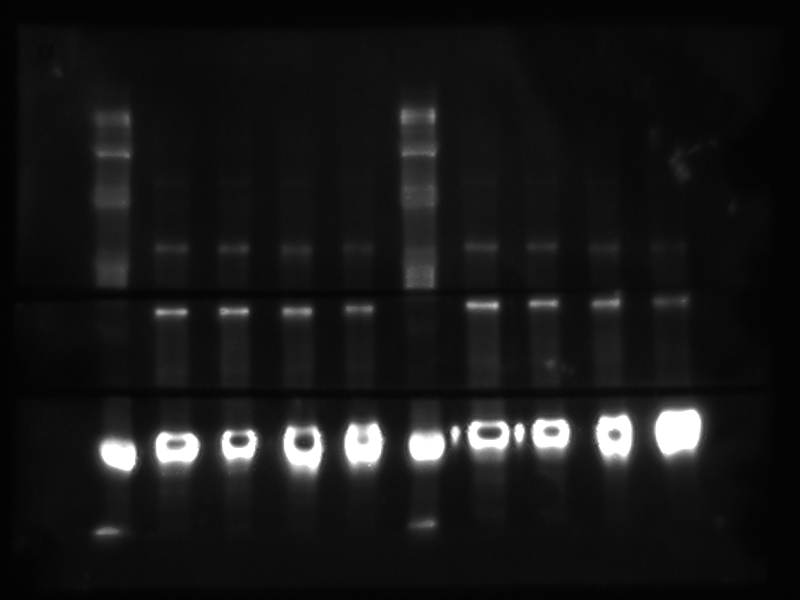

Supplement: S1 File — (ZIP) [file pone.0170762.s011.zip › immunoblot_analysis/images/170714_DuploBlot5_2.5min.tiff]

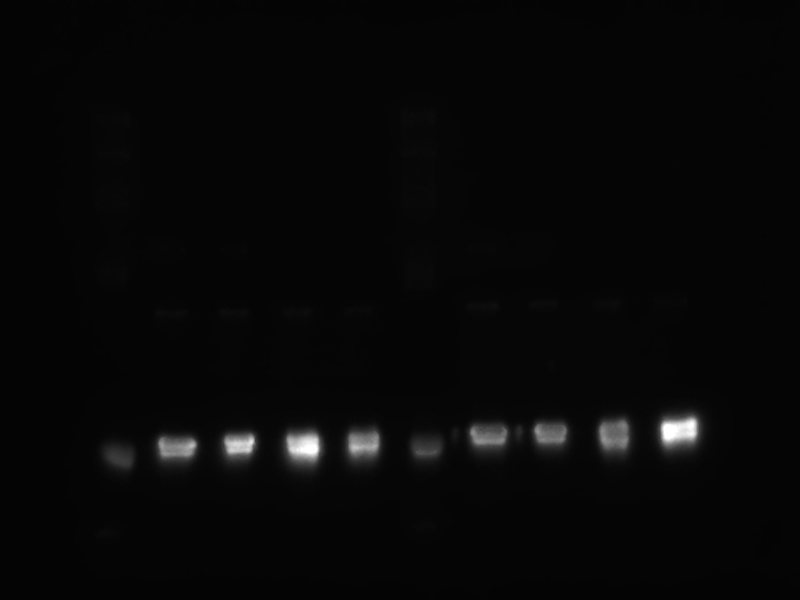

Supplement: S1 File — (ZIP) [file pone.0170762.s011.zip › immunoblot_analysis/images/170714_DuploBlot5_5sec.tiff]

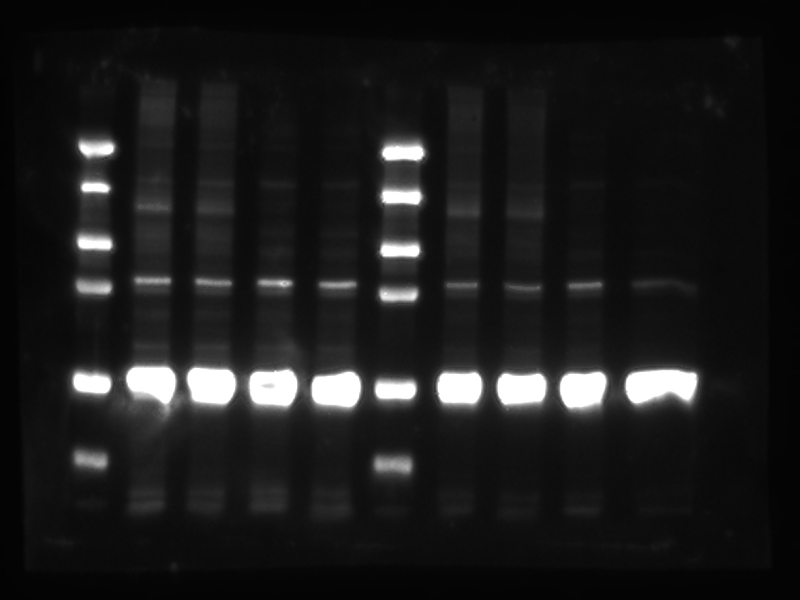

Supplement: S1 File — (ZIP) [file pone.0170762.s011.zip › immunoblot_analysis/images/210214_DuploBlot1_2min.tiff]

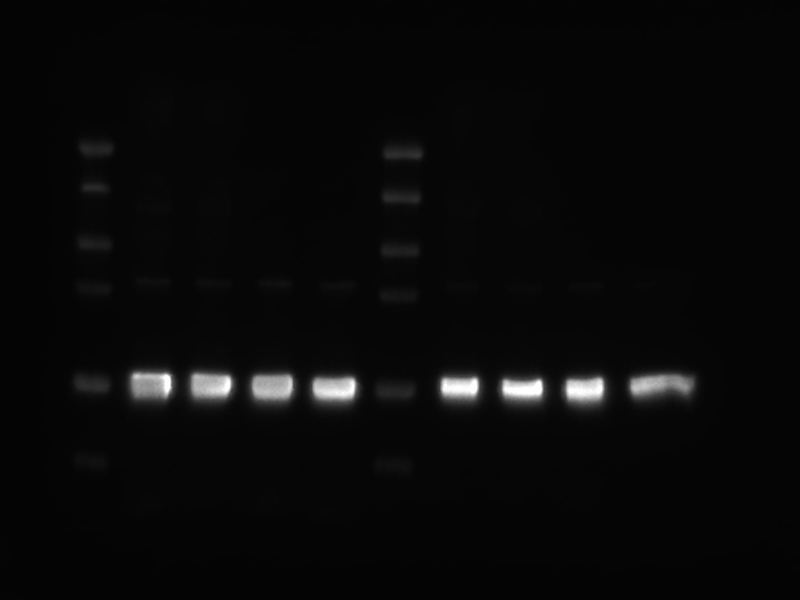

Supplement: S1 File — (ZIP) [file pone.0170762.s011.zip › immunoblot_analysis/images/210214_DuploBlot1_5sec.tiff]

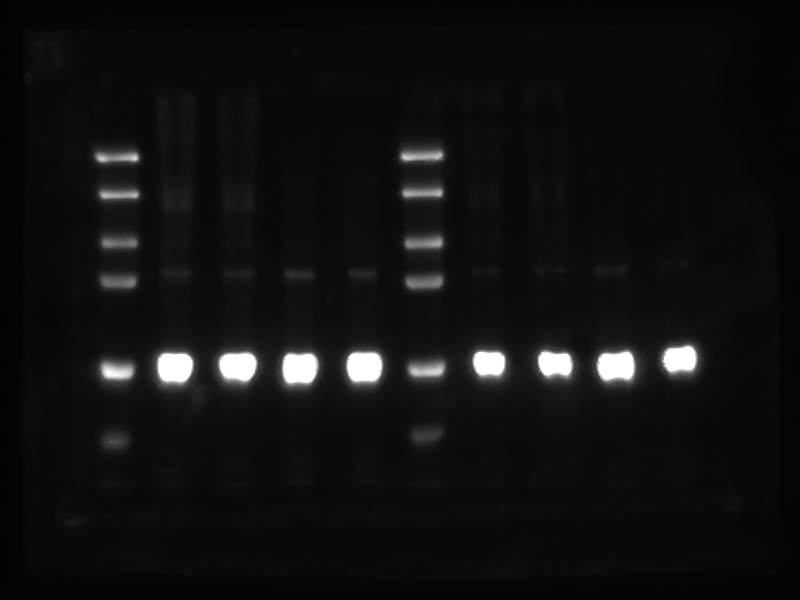

Supplement: S1 File — (ZIP) [file pone.0170762.s011.zip › immunoblot_analysis/images/210214_DuploBlot2_2min.tiff]

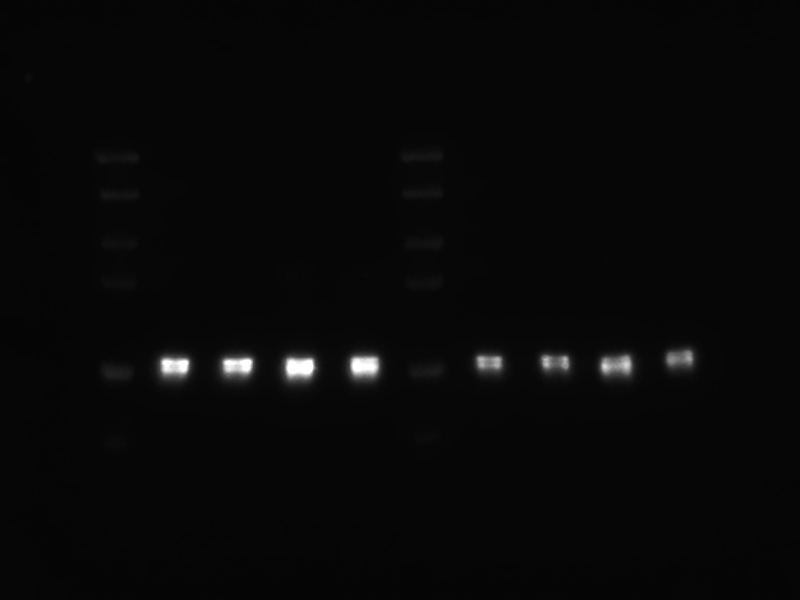

Supplement: S1 File — (ZIP) [file pone.0170762.s011.zip › immunoblot_analysis/images/210214_DuploBlot2_5sec.tiff]

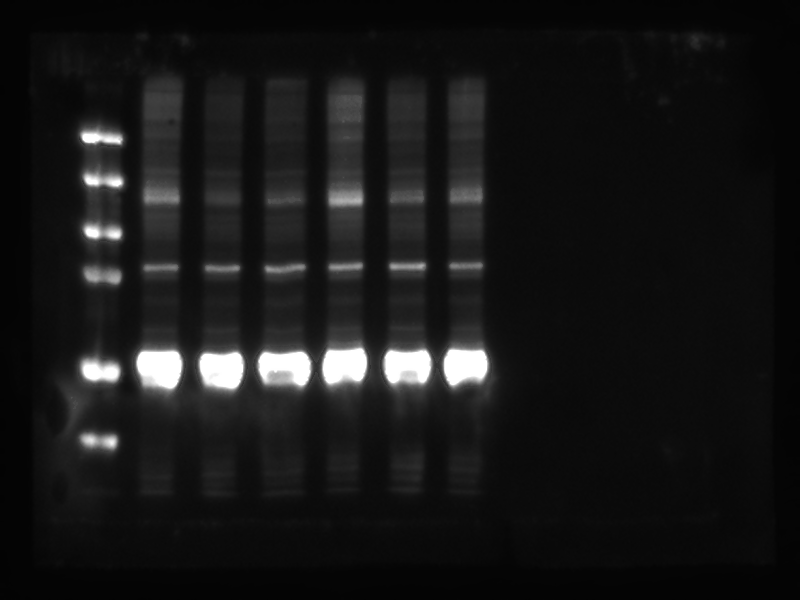

Supplement: S1 File — (ZIP) [file pone.0170762.s011.zip › immunoblot_analysis/images/210214_MSblot1_2min.tiff]

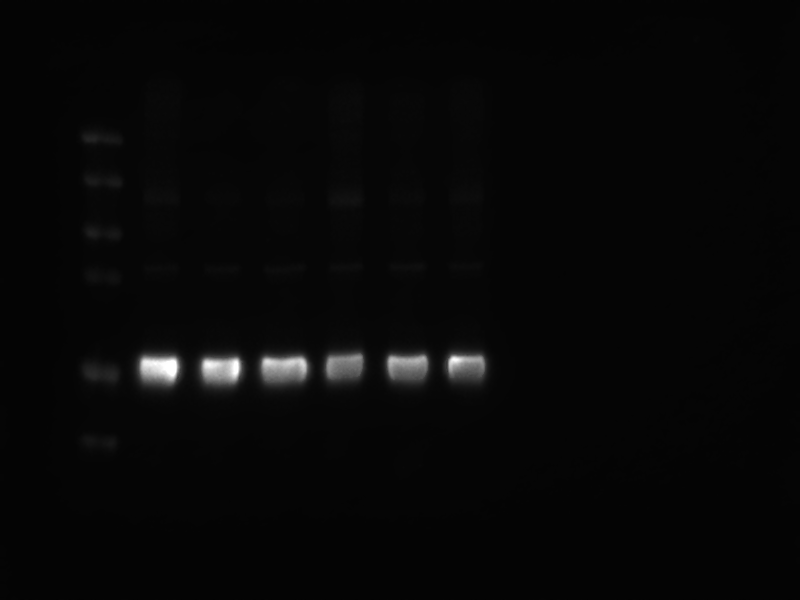

Supplement: S1 File — (ZIP) [file pone.0170762.s011.zip › immunoblot_analysis/images/210214_MSblot1_5sec.tiff]

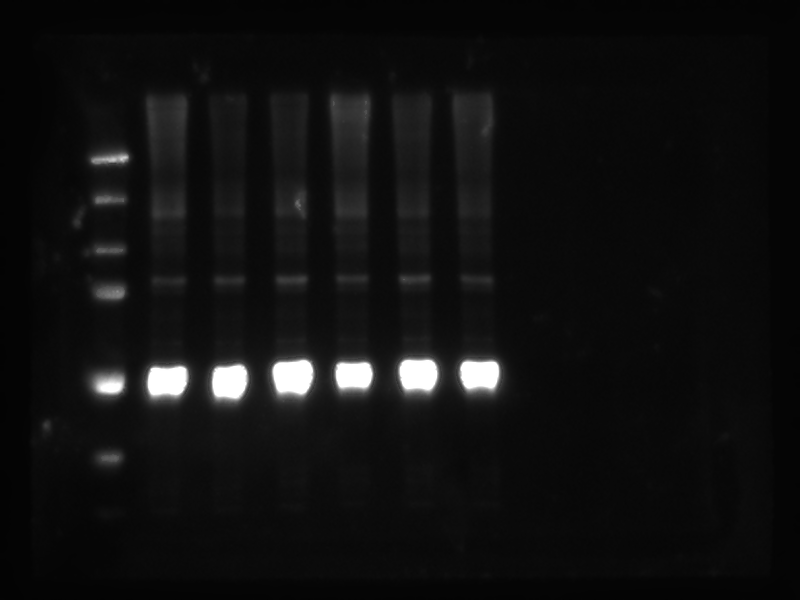

Supplement: S1 File — (ZIP) [file pone.0170762.s011.zip › immunoblot_analysis/images/210214_MSblot2_2min.tiff]

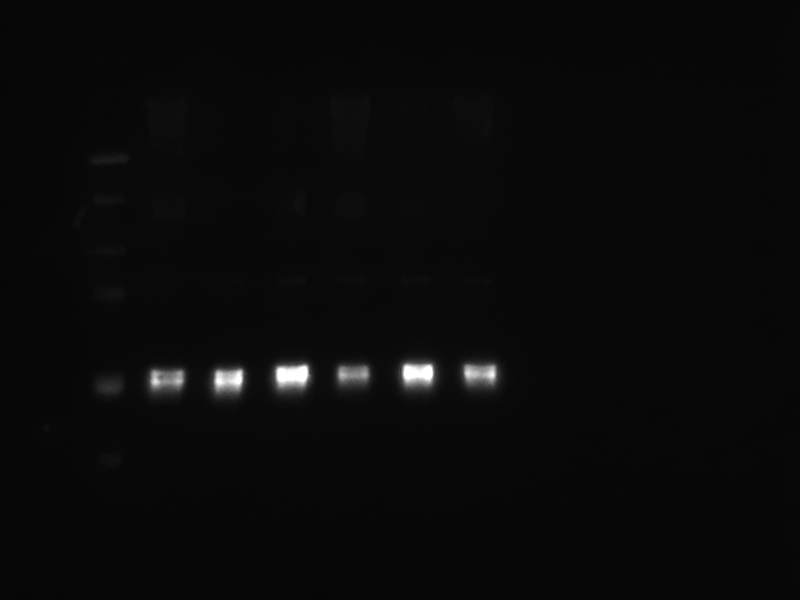

Supplement: S1 File — (ZIP) [file pone.0170762.s011.zip › immunoblot_analysis/images/210214_MSblot2_5sec.tiff]

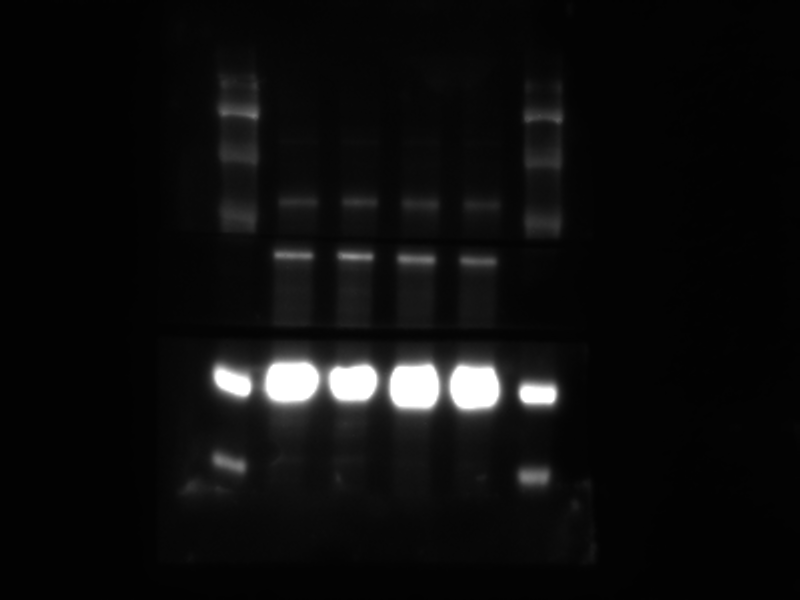

Supplement: S1 File — (ZIP) [file pone.0170762.s011.zip › immunoblot_analysis/images/280514_DuploBlot3_2.5min.tiff]

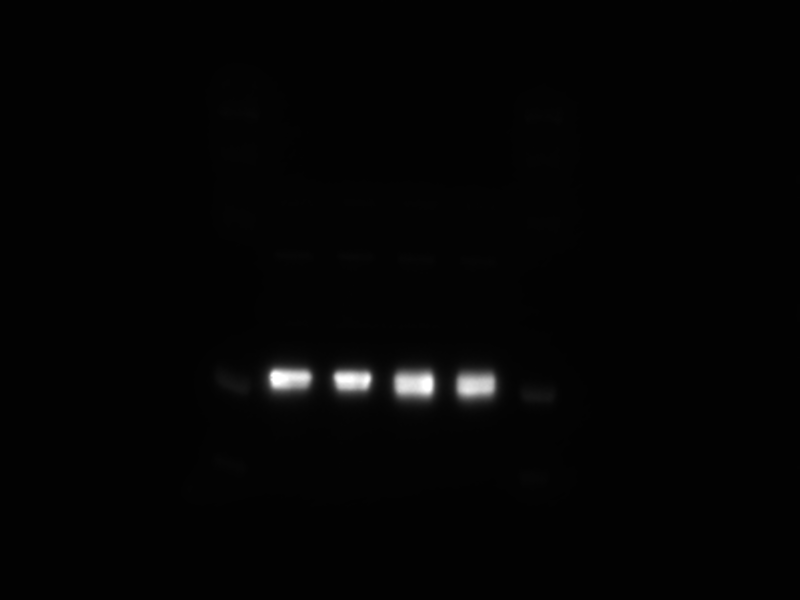

Supplement: S1 File — (ZIP) [file pone.0170762.s011.zip › immunoblot_analysis/images/280514_DuploBlot3_5sec.tiff]

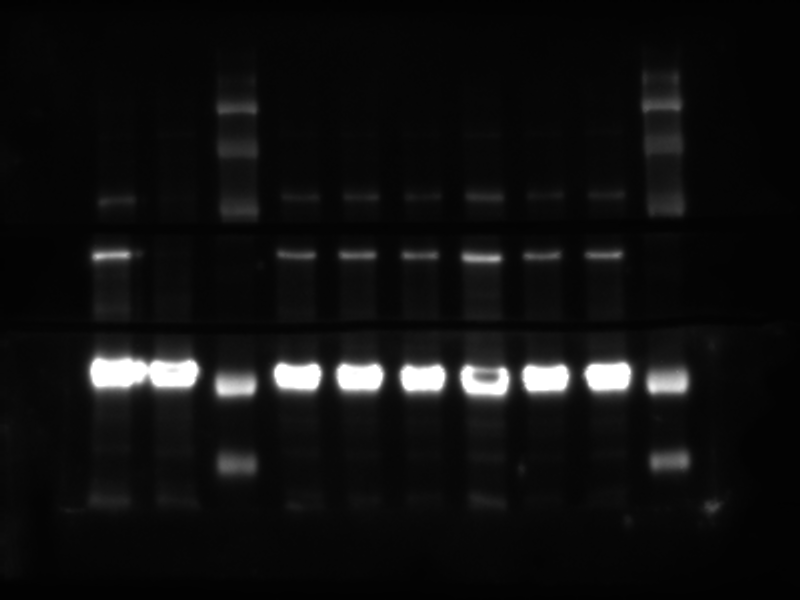

Supplement: S1 File — (ZIP) [file pone.0170762.s011.zip › immunoblot_analysis/images/280514_MSblot5_2.5min.tiff]

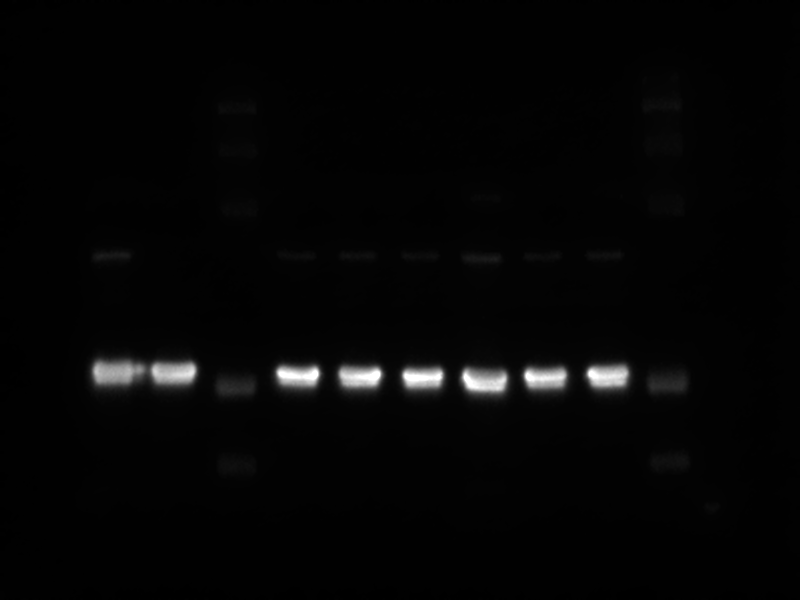

Supplement: S1 File — (ZIP) [file pone.0170762.s011.zip › immunoblot_analysis/images/280514_MSblot5_5sec.tiff]
