# Supplementary material for: Semi-quantitative proteomics of mammalian cells upon short-term exposure to non-ionizing electromagnetic fields
Source: PLoS One. 2017 Feb 24;12(2):e0170762. doi: 10.1371/journal.pone.0170762 (PMC5325209; doi:10.1371/journal.pone.0170762)
Supplement: S5 Table — This table was obtained by querying the EMF databases via the PIQMIe service (refer to the 'Search Grid' tab). Note that the leading proteins (accessions) of non-singleton groups are in red; normalized SILAC protein ratios: H/L, H/M, M/L including their reciprocals; sequence coverage (SeqCov) expressed as the number of amino acids spanned by the quantitated peptides divided by the sequence length of the leading protein; the number of peptide quantifications (Nq), standard deviation of the mean log2-transformed peptide ratios (SD) and the peak intensity-base significance B (SigB, p-values adjusted by the Benjamini-Hochberg method). (DOC) [file pone.0170762.s010.doc]

**S5 Table.**

| **ELF** | | | | | | | | | | | | | | | | | | | | | | | | |
| --- | --- | --- | --- | --- | --- | --- | --- | --- | --- | --- | --- | --- | --- | --- | --- | --- | --- | --- | --- | --- | --- | --- | --- | --- |
| **GroupID** | **Size** | **Experiment** | **Protein accessions** | **Protein names** | **Evidence** | **Genes** | **Organism** | **PEP** | **H/L** | **L/H** | **H/M** | **M/H** | **M/L** | **L/M** | **SeqCov** | **Nq HL** | **Nq HM** | **Nq ML** | **SD HL** | **SD HM** | **SD ML** | **SigB HL** | **SigB HM** | **SigB ML** |
| 1871 | 8 | VH10_L0_M0_H1 | **UniProtKB/Swiss-Prot**: [P40692](http://www.uniprot.org/uniprot/P40692) [P40692-3](http://www.uniprot.org/uniprot/P40692-3) [P40692-2](http://www.uniprot.org/uniprot/P40692-2)   **UniProtKB/TrEMBL**: [H0Y818](http://www.uniprot.org/uniprot/H0Y818) [A0A087WX20](http://www.uniprot.org/uniprot/A0A087WX20) [H0Y806](http://www.uniprot.org/uniprot/H0Y806) [E9PF25](http://www.uniprot.org/uniprot/E9PF25) [F2Z298](http://www.uniprot.org/uniprot/F2Z298) | DNA mismatch repair protein Mlh1 | protein | MLH1 | Homo sapiens | 9.3484e-109 | 2.81 | 0.36 | 2.71 | 0.37 | 1.04 | 0.96 | 2.65 | 2 | 2 | 2 | 2.09 | 2.19 | 0.10 | 0.0000 | 0.0000 | 0.8706 |
| 1871 | 8 | VH10_L1_M1_H0 | **UniProtKB/Swiss-Prot**: [P40692](http://www.uniprot.org/uniprot/P40692) [P40692-3](http://www.uniprot.org/uniprot/P40692-3) [P40692-2](http://www.uniprot.org/uniprot/P40692-2)   **UniProtKB/TrEMBL**: [H0Y818](http://www.uniprot.org/uniprot/H0Y818) [A0A087WX20](http://www.uniprot.org/uniprot/A0A087WX20) [H0Y806](http://www.uniprot.org/uniprot/H0Y806) [E9PF25](http://www.uniprot.org/uniprot/E9PF25) [F2Z298](http://www.uniprot.org/uniprot/F2Z298) | DNA mismatch repair protein Mlh1 | protein | MLH1 | Homo sapiens | 9.3484e-109 | 0.61 | 1.63 | 0.55 | 1.82 | 1.10 | 0.91 | 1.85 | 2 | 2 | 2 | 0.31 | 0.08 | 0.23 | 0.0523 | 0.0056 | 0.9086 |
| 2824 | 1 | VH10_L0_M0_H1 | **UniProtKB/Swiss-Prot**: [Q14691](http://www.uniprot.org/uniprot/Q14691) | DNA replication complex GINS protein PSF1 | protein | GINS1 | Homo sapiens | 4.7817e-38 | 1.19 | 0.84 | 1.24 | 0.81 | 0.99 | 1.01 | 11.22 | 3 | 3 | 3 | 0.12 | 0.24 | 0.04 | 0.4204 | 0.0856 | 0.9945 |
| 2824 | 1 | VH10_L1_M1_H0 | **UniProtKB/Swiss-Prot**: [Q14691](http://www.uniprot.org/uniprot/Q14691) | DNA replication complex GINS protein PSF1 | protein | GINS1 | Homo sapiens | 4.7817e-38 | 0.79 | 1.26 | 0.80 | 1.25 | 1.04 | 0.96 | 11.22 | 3 | 3 | 3 | 0.20 | 0.21 | 0.04 | 0.1085 | 0.0332 | 0.8804 |
| 2013 | 3 | VH10_L0_M0_H1 | **UniProtKB/Swiss-Prot**: [P49459](http://www.uniprot.org/uniprot/P49459) [P49459-3](http://www.uniprot.org/uniprot/P49459-3) [P49459-2](http://www.uniprot.org/uniprot/P49459-2) | Ubiquitin-conjugating enzyme E2 A | protein | UBE2A | Homo sapiens | 6.4972e-49 | 1.30 | 0.77 | 1.24 | 0.81 | 1.04 | 0.96 | 24.34 | 2 | 2 | 2 | 0.40 | 0.43 | 0.00 | 0.2963 | 0.1697 | 0.8230 |
| 2013 | 3 | VH10_L1_M1_H0 | **UniProtKB/Swiss-Prot**: [P49459](http://www.uniprot.org/uniprot/P49459) [P49459-3](http://www.uniprot.org/uniprot/P49459-3) [P49459-2](http://www.uniprot.org/uniprot/P49459-2) | Ubiquitin-conjugating enzyme E2 A | protein | UBE2A | Homo sapiens | 6.4972e-49 | 0.70 | 1.43 | 0.86 | 1.17 | 0.82 | 1.23 | 18.42 | 2 | 2 | 2 | 0.31 | 0.02 | 0.29 | 0.1473 | 0.2519 | 0.3392 |
| 3524 | 1 | VH10_L0_M0_H1 | **UniProtKB/Swiss-Prot**: [Q8IWA0](http://www.uniprot.org/uniprot/Q8IWA0) | WD repeat-containing protein 75 | protein | WDR75 | Homo sapiens | 1.4234e-87 | 1.30 | 0.77 | 1.23 | 0.81 | 1.00 | 1.00 | 6.63 | 4 | 4 | 4 | 0.19 | 0.39 | 0.45 | 0.2964 | 0.1793 | 0.9547 |
| 3524 | 1 | VH10_L1_M1_H0 | **UniProtKB/Swiss-Prot**: [Q8IWA0](http://www.uniprot.org/uniprot/Q8IWA0) | WD repeat-containing protein 75 | protein | WDR75 | Homo sapiens | 1.4234e-87 | 0.78 | 1.29 | 0.68 | 1.46 | 1.12 | 0.90 | 3.98 | 3 | 3 | 3 | 0.20 | 0.16 | 0.10 | 0.2881 | 0.0074 | 0.7580 |
| 3519 | 2 | VH10_L0_M0_H1 | **UniProtKB/Swiss-Prot**: [Q8IVL6](http://www.uniprot.org/uniprot/Q8IVL6) [Q8IVL6-2](http://www.uniprot.org/uniprot/Q8IVL6-2) | Prolyl 3-hydroxylase 3 | protein | LEPREL2 | Homo sapiens | 3.3331e-65 | 1.90 | 0.53 | 2.26 | 0.44 | 0.94 | 1.06 | 2.85 | 3 | 3 | 3 | 0.68 | 1.05 | 0.39 | 0.0063 | 0.0000 | 0.7764 |
| 3519 | 2 | VH10_L1_M1_H0 | **UniProtKB/Swiss-Prot**: [Q8IVL6](http://www.uniprot.org/uniprot/Q8IVL6) [Q8IVL6-2](http://www.uniprot.org/uniprot/Q8IVL6-2) | Prolyl 3-hydroxylase 3 | protein | LEPREL2 | Homo sapiens | 3.3331e-65 | 0.86 | 1.17 | 0.85 | 1.18 | 1.00 | 1.00 | 11.01 | 6 | 6 | 7 | 0.17 | 0.30 | 0.79 | 0.2100 | 0.0539 | 0.9826 |
| 2029 | 14 | VH10_L0_M0_H1 | **UniProtKB/Swiss-Prot**: [P49757](http://www.uniprot.org/uniprot/P49757) [P49757-3](http://www.uniprot.org/uniprot/P49757-3) [P49757-2](http://www.uniprot.org/uniprot/P49757-2) [P49757-4](http://www.uniprot.org/uniprot/P49757-4) [P49757-5](http://www.uniprot.org/uniprot/P49757-5) [P49757-6](http://www.uniprot.org/uniprot/P49757-6) [P49757-7](http://www.uniprot.org/uniprot/P49757-7) [P49757-8](http://www.uniprot.org/uniprot/P49757-8) [P49757-9](http://www.uniprot.org/uniprot/P49757-9)   **UniProtKB/TrEMBL**: [G3V3Z8](http://www.uniprot.org/uniprot/G3V3Z8) [G3V3R1](http://www.uniprot.org/uniprot/G3V3R1) [G3V3M5](http://www.uniprot.org/uniprot/G3V3M5) [G3V433](http://www.uniprot.org/uniprot/G3V433) [G3V4S6](http://www.uniprot.org/uniprot/G3V4S6) | Protein numb homolog | protein | NUMB | Homo sapiens | 6.0502e-7 | 1.16 | 0.86 | 1.17 | 0.85 | 0.99 | 1.01 | 1.69 | 2 | 2 | 2 | 0.12 | 0.11 | 0.01 | 0.6195 | 0.4954 | 0.9544 |
| 2029 | 14 | VH10_L1_M1_H0 | **UniProtKB/Swiss-Prot**: [P49757](http://www.uniprot.org/uniprot/P49757) [P49757-3](http://www.uniprot.org/uniprot/P49757-3) [P49757-2](http://www.uniprot.org/uniprot/P49757-2) [P49757-4](http://www.uniprot.org/uniprot/P49757-4) [P49757-5](http://www.uniprot.org/uniprot/P49757-5) [P49757-6](http://www.uniprot.org/uniprot/P49757-6) [P49757-7](http://www.uniprot.org/uniprot/P49757-7) [P49757-8](http://www.uniprot.org/uniprot/P49757-8) [P49757-9](http://www.uniprot.org/uniprot/P49757-9)   **UniProtKB/TrEMBL**: [G3V3Z8](http://www.uniprot.org/uniprot/G3V3Z8) [G3V3R1](http://www.uniprot.org/uniprot/G3V3R1) [G3V3M5](http://www.uniprot.org/uniprot/G3V3M5) [G3V433](http://www.uniprot.org/uniprot/G3V433) [G3V4S6](http://www.uniprot.org/uniprot/G3V4S6) | Protein numb homolog | protein | NUMB | Homo sapiens | 6.0502e-7 | 0.58 | 1.72 | 0.62 | 1.63 | 0.94 | 1.06 | 2.30 | 2 | 2 | 2 | 0.05 | 0.01 | 0.06 | 0.0324 | 0.0244 | 0.6514 |
| 4558 | 4 | U2OS_L0_M0_H1 | **UniProtKB/Swiss-Prot**: [Q9H6R0](http://www.uniprot.org/uniprot/Q9H6R0) [Q9H6R0-2](http://www.uniprot.org/uniprot/Q9H6R0-2)   **UniProtKB/TrEMBL**: [I3L1L6](http://www.uniprot.org/uniprot/I3L1L6) [Q05BE5](http://www.uniprot.org/uniprot/Q05BE5) | DEAH (Asp-Glu-Ala-His) box polypeptide 33, Putative ATP-dependent RNA helicase DHX33 | protein | DHX33 | Homo sapiens | 6.7687e-18 | 1.19 | 0.84 | 1.27 | 0.79 | 0.92 | 1.08 | 3.11 | 2 | 2 | 2 | 0.13 | 0.16 | 0.03 | 0.4868 | 0.1988 | 0.7826 |
| 4558 | 4 | U2OS_L1_M1_H0 | **UniProtKB/Swiss-Prot**: [Q9H6R0](http://www.uniprot.org/uniprot/Q9H6R0) [Q9H6R0-2](http://www.uniprot.org/uniprot/Q9H6R0-2)   **UniProtKB/TrEMBL**: [I3L1L6](http://www.uniprot.org/uniprot/I3L1L6) [Q05BE5](http://www.uniprot.org/uniprot/Q05BE5) | DEAH (Asp-Glu-Ala-His) box polypeptide 33, Putative ATP-dependent RNA helicase DHX33 | protein | DHX33 | Homo sapiens | 6.7687e-18 | 0.76 | 1.32 | 0.70 | 1.43 | 1.09 | 0.92 | 5.23 | 2 | 2 | 2 | 0.67 | 0.54 | 0.15 | 0.2325 | 0.0674 | 0.7319 |
| 2508 | 6 | U2OS_L0_M0_H1 | **UniProtKB/Swiss-Prot**: [Q03426](http://www.uniprot.org/uniprot/Q03426)   **UniProtKB/TrEMBL**: [F5H8H2](http://www.uniprot.org/uniprot/F5H8H2) [F5H092](http://www.uniprot.org/uniprot/F5H092) [F5GXC0](http://www.uniprot.org/uniprot/F5GXC0) [F5H368](http://www.uniprot.org/uniprot/F5H368) [F5H163](http://www.uniprot.org/uniprot/F5H163) | Mevalonate kinase | protein | MVK | Homo sapiens | 8.4081e-88 | 1.62 | 0.62 | 1.36 | 0.74 | 1.08 | 0.93 | 14.90 | 5 | 5 | 5 | 0.48 | 0.38 | 0.16 | 0.0200 | 0.0439 | 0.6206 |
| 2593 | 4 | U2OS_L0_M0_H1 | **UniProtKB/Swiss-Prot**: [Q12788](http://www.uniprot.org/uniprot/Q12788)   **UniProtKB/TrEMBL**: [J3KNP2](http://www.uniprot.org/uniprot/J3KNP2) [A0A087WYP7](http://www.uniprot.org/uniprot/A0A087WYP7) [H3BN88](http://www.uniprot.org/uniprot/H3BN88) | Transducin beta-like protein 3 | protein | TBL3 | Homo sapiens | 4.4851e-232 | 1.14 | 0.88 | 1.20 | 0.83 | 0.90 | 1.11 | 10.77 | 9 | 9 | 9 | 0.62 | 0.42 | 0.42 | 0.2758 | 0.0297 | 0.3468 |
| 2593 | 4 | U2OS_L1_M1_H0 | **UniProtKB/Swiss-Prot**: [Q12788](http://www.uniprot.org/uniprot/Q12788)   **UniProtKB/TrEMBL**: [J3KNP2](http://www.uniprot.org/uniprot/J3KNP2) [A0A087WYP7](http://www.uniprot.org/uniprot/A0A087WYP7) [H3BN88](http://www.uniprot.org/uniprot/H3BN88) | Transducin beta-like protein 3 | protein | TBL3 | Homo sapiens | 4.4851e-232 | 0.81 | 1.23 | 0.74 | 1.36 | 0.95 | 1.05 | 15.97 | 12 | 12 | 12 | 0.47 | 0.39 | 0.30 | 0.0751 | 0.0003 | 0.7012 |
| 5002 | 2 | U2OS_L0_M0_H1 | **UniProtKB/Swiss-Prot**: [Q9UGP4](http://www.uniprot.org/uniprot/Q9UGP4)   **UniProtKB/TrEMBL**: [C9JRJ5](http://www.uniprot.org/uniprot/C9JRJ5) | LIM domain-containing protein 1 | protein | LIMD1 | Homo sapiens | 8.5776e-37 | 0.62 | 1.60 | 0.65 | 1.53 | 0.95 | 1.05 | 4.88 | 3 | 3 | 3 | 0.30 | 0.32 | 0.39 | 0.0656 | 0.0253 | 0.8860 |
| 5002 | 2 | U2OS_L1_M1_H0 | **UniProtKB/Swiss-Prot**: [Q9UGP4](http://www.uniprot.org/uniprot/Q9UGP4)   **UniProtKB/TrEMBL**: [C9JRJ5](http://www.uniprot.org/uniprot/C9JRJ5) | LIM domain-containing protein 1 | protein | LIMD1 | Homo sapiens | 8.5776e-37 | 1.11 | 0.90 | 1.06 | 0.94 | 0.98 | 1.02 | 4.29 | 4 | 4 | 4 | 0.44 | 0.39 | 0.15 | 0.8328 | 0.8421 | 0.8586 |
| 2008 | 9 | U2OS_L0_M0_H1 | **UniProtKB/Swiss-Prot**: [P49418](http://www.uniprot.org/uniprot/P49418) [P49418-2](http://www.uniprot.org/uniprot/P49418-2) [Q9UBW5](http://www.uniprot.org/uniprot/Q9UBW5) [Q9UBW5-2](http://www.uniprot.org/uniprot/Q9UBW5-2)   **UniProtKB/TrEMBL**: [H0Y7T8](http://www.uniprot.org/uniprot/H0Y7T8) [A0A087X188](http://www.uniprot.org/uniprot/A0A087X188) [A0A0A0MT10](http://www.uniprot.org/uniprot/A0A0A0MT10) [F5H0W4](http://www.uniprot.org/uniprot/F5H0W4) [S4R418](http://www.uniprot.org/uniprot/S4R418) | Bridging integrator 2, Amphiphysin | protein | AMPH, BIN2 | Homo sapiens | 2.3937e-100 | 0.76 | 1.31 | 0.68 | 1.47 | 1.29 | 0.77 | 7.34 | 4 | 4 | 4 | 0.23 | 0.42 | 0.26 | 0.3129 | 0.0420 | 0.2518 |
| 2008 | 9 | U2OS_L1_M1_H0 | **UniProtKB/Swiss-Prot**: [P49418](http://www.uniprot.org/uniprot/P49418) [P49418-2](http://www.uniprot.org/uniprot/P49418-2) [Q9UBW5](http://www.uniprot.org/uniprot/Q9UBW5) [Q9UBW5-2](http://www.uniprot.org/uniprot/Q9UBW5-2)   **UniProtKB/TrEMBL**: [H0Y7T8](http://www.uniprot.org/uniprot/H0Y7T8) [A0A087X188](http://www.uniprot.org/uniprot/A0A087X188) [A0A0A0MT10](http://www.uniprot.org/uniprot/A0A0A0MT10) [F5H0W4](http://www.uniprot.org/uniprot/F5H0W4) [S4R418](http://www.uniprot.org/uniprot/S4R418) | Bridging integrator 2, Amphiphysin | protein | AMPH, BIN2 | Homo sapiens | 2.3937e-100 | 1.53 | 0.65 | 1.53 | 0.65 | 1.00 | 1.00 | 4.32 | 2 | 2 | 2 | 1.01 | 1.04 | 0.02 | 0.2019 | 0.0729 | 0.9783 |
| 1649 | 7 | U2OS_L0_M0_H1 | **UniProtKB/Swiss-Prot**: [P26358](http://www.uniprot.org/uniprot/P26358) [P26358-2](http://www.uniprot.org/uniprot/P26358-2) [P26358-3](http://www.uniprot.org/uniprot/P26358-3)   **UniProtKB/TrEMBL**: [K7ENW7](http://www.uniprot.org/uniprot/K7ENW7) [K7EJL0](http://www.uniprot.org/uniprot/K7EJL0) [K7EMU8](http://www.uniprot.org/uniprot/K7EMU8) [K7EIZ6](http://www.uniprot.org/uniprot/K7EIZ6) | DNA (cytosine-5)-methyltransferase 1 | protein | DNMT1 | Homo sapiens | 6.5935e-185 | 0.72 | 1.38 | 0.69 | 1.45 | 0.82 | 1.23 | 1.72 | 3 | 3 | 3 | 0.59 | 0.75 | 0.74 | 0.2197 | 0.0535 | 0.3904 |
| 1649 | 7 | U2OS_L1_M1_H0 | **UniProtKB/Swiss-Prot**: [P26358](http://www.uniprot.org/uniprot/P26358) [P26358-2](http://www.uniprot.org/uniprot/P26358-2) [P26358-3](http://www.uniprot.org/uniprot/P26358-3)   **UniProtKB/TrEMBL**: [K7ENW7](http://www.uniprot.org/uniprot/K7ENW7) [K7EJL0](http://www.uniprot.org/uniprot/K7EJL0) [K7EMU8](http://www.uniprot.org/uniprot/K7EMU8) [K7EIZ6](http://www.uniprot.org/uniprot/K7EIZ6) | DNA (cytosine-5)-methyltransferase 1 | protein | DNMT1 | Homo sapiens | 6.5935e-185 | 1.43 | 0.70 | 1.30 | 0.77 | 1.09 | 0.91 | 1.29 | 2 | 2 | 2 | 0.55 | 1.20 | 0.65 | 0.2946 | 0.2948 | 0.7211 |
| 2061 | 10 | U2OS_L0_M0_H1 | **UniProtKB/Swiss-Prot**: [P50579](http://www.uniprot.org/uniprot/P50579) [P50579-2](http://www.uniprot.org/uniprot/P50579-2) [P50579-3](http://www.uniprot.org/uniprot/P50579-3)   **UniProtKB/TrEMBL**: [F8VQZ7](http://www.uniprot.org/uniprot/F8VQZ7) [A0A0A6YYK3](http://www.uniprot.org/uniprot/A0A0A6YYK3) [F8VRR3](http://www.uniprot.org/uniprot/F8VRR3) [G3V1U3](http://www.uniprot.org/uniprot/G3V1U3) [F8VY03](http://www.uniprot.org/uniprot/F8VY03) [F8VSC4](http://www.uniprot.org/uniprot/F8VSC4) [F8VZX9](http://www.uniprot.org/uniprot/F8VZX9) | Methionine aminopeptidase 2 | protein | METAP2 | Homo sapiens | 0e+0 | 0.68 | 1.47 | 0.85 | 1.17 | 0.84 | 1.20 | 16.32 | 5 | 5 | 5 | 0.12 | 0.07 | 0.18 | 0.0502 | 0.2153 | 0.3379 |
| 2061 | 10 | U2OS_L1_M1_H0 | **UniProtKB/Swiss-Prot**: [P50579](http://www.uniprot.org/uniprot/P50579) [P50579-2](http://www.uniprot.org/uniprot/P50579-2) [P50579-3](http://www.uniprot.org/uniprot/P50579-3)   **UniProtKB/TrEMBL**: [F8VQZ7](http://www.uniprot.org/uniprot/F8VQZ7) [A0A0A6YYK3](http://www.uniprot.org/uniprot/A0A0A6YYK3) [F8VRR3](http://www.uniprot.org/uniprot/F8VRR3) [G3V1U3](http://www.uniprot.org/uniprot/G3V1U3) [F8VY03](http://www.uniprot.org/uniprot/F8VY03) [F8VSC4](http://www.uniprot.org/uniprot/F8VSC4) [F8VZX9](http://www.uniprot.org/uniprot/F8VZX9) | Methionine aminopeptidase 2 | protein | METAP2 | Homo sapiens | 0e+0 | 1.35 | 0.74 | 1.33 | 0.75 | 1.01 | 0.99 | 10.04 | 2 | 2 | 2 | 0.63 | 0.63 | 0.01 | 0.3898 | 0.2500 | 0.9613 |
| 2038 | 6 | U2OS_L0_M0_H1 | **UniProtKB/Swiss-Prot**: [P49902](http://www.uniprot.org/uniprot/P49902) [P49902-2](http://www.uniprot.org/uniprot/P49902-2)   **UniProtKB/TrEMBL**: [Q5JUV4](http://www.uniprot.org/uniprot/Q5JUV4) [Q5JUV3](http://www.uniprot.org/uniprot/Q5JUV3) [Q5JUV6](http://www.uniprot.org/uniprot/Q5JUV6) [H0YHR8](http://www.uniprot.org/uniprot/H0YHR8) | Cytosolic purine 5'-nucleotidase | protein | NT5C2 | Homo sapiens | 3.0204e-76 | 0.80 | 1.24 | 0.85 | 1.18 | 0.88 | 1.14 | 6.77 | 5 | 5 | 5 | 0.22 | 0.15 | 0.33 | 0.0068 | 0.0014 | 0.1148 |
| 2818 | 4 | IB10_L0_M0_H1 | **UniProtKB/Swiss-Prot**: [Q8BZM1](http://www.uniprot.org/uniprot/Q8BZM1)   **UniProtKB/TrEMBL**: [Q3T9A5](http://www.uniprot.org/uniprot/Q3T9A5) [D3Z0L3](http://www.uniprot.org/uniprot/D3Z0L3) [D6RGR3](http://www.uniprot.org/uniprot/D6RGR3) | Glomulin | transcript | Glmn | Mus musculus | 1.7075e-79 | 2.00 | 0.50 | 1.62 | 0.62 | 0.85 | 1.17 | 5.20 | 3 | 3 | 3 | 0.57 | 0.57 | 0.24 | 0.0202 | 0.0617 | 0.3896 |
| 2818 | 4 | IB10_L1_M1_H0 | **UniProtKB/Swiss-Prot**: [Q8BZM1](http://www.uniprot.org/uniprot/Q8BZM1)   **UniProtKB/TrEMBL**: [Q3T9A5](http://www.uniprot.org/uniprot/Q3T9A5) [D3Z0L3](http://www.uniprot.org/uniprot/D3Z0L3) [D6RGR3](http://www.uniprot.org/uniprot/D6RGR3) | Glomulin | transcript | Glmn | Mus musculus | 1.7075e-79 | 0.74 | 1.35 | 0.85 | 1.18 | 0.92 | 1.09 | 6.04 | 3 | 3 | 3 | 0.91 | 1.32 | 0.46 | 0.2043 | 0.3597 | 0.7778 |
| 2586 | 6 | IB10_L0_M0_H1 | **UniProtKB/Swiss-Prot**: [Q8BG51-4](http://www.uniprot.org/uniprot/Q8BG51-4) [Q8BG51-3](http://www.uniprot.org/uniprot/Q8BG51-3) [Q8BG51-2](http://www.uniprot.org/uniprot/Q8BG51-2) [Q8BG51](http://www.uniprot.org/uniprot/Q8BG51) [Q8JZN7](http://www.uniprot.org/uniprot/Q8JZN7)   **UniProtKB/TrEMBL**: [F7ASU3](http://www.uniprot.org/uniprot/F7ASU3) | Mitochondrial Rho GTPase 2, Mitochondrial Rho GTPase 1 | transcript | Rhot1, Rhot2 | Mus musculus | 1.5048e-7 | 1.37 | 0.73 | 1.25 | 0.80 | 1.12 | 0.89 | 1.42 | 2 | 2 | 2 | 0.43 | 0.58 | 0.15 | 0.3406 | 0.4344 | 0.7427 |
| 2586 | 6 | IB10_L1_M1_H0 | **UniProtKB/Swiss-Prot**: [Q8BG51-4](http://www.uniprot.org/uniprot/Q8BG51-4) [Q8BG51-3](http://www.uniprot.org/uniprot/Q8BG51-3) [Q8BG51-2](http://www.uniprot.org/uniprot/Q8BG51-2) [Q8BG51](http://www.uniprot.org/uniprot/Q8BG51) [Q8JZN7](http://www.uniprot.org/uniprot/Q8JZN7)   **UniProtKB/TrEMBL**: [F7ASU3](http://www.uniprot.org/uniprot/F7ASU3) | Mitochondrial Rho GTPase 2, Mitochondrial Rho GTPase 1 | transcript | Rhot1, Rhot2 | Mus musculus | 1.5048e-7 | 0.83 | 1.21 | 0.74 | 1.35 | 1.11 | 0.90 | 2.98 | 2 | 2 | 2 | 0.01 | 0.26 | 0.27 | 0.4532 | 0.0699 | 0.5070 |
| 3445 | 6 | IB10_L0_M0_H1 | **UniProtKB/Swiss-Prot**: [Q921W4](http://www.uniprot.org/uniprot/Q921W4) [Q921W4-2](http://www.uniprot.org/uniprot/Q921W4-2)   **UniProtKB/TrEMBL**: [D3Z6I4](http://www.uniprot.org/uniprot/D3Z6I4) [D3YZD6](http://www.uniprot.org/uniprot/D3YZD6) [F7BGV1](http://www.uniprot.org/uniprot/F7BGV1) [D3YU21](http://www.uniprot.org/uniprot/D3YU21) | Quinone oxidoreductase-like protein 1, Crystallin, zeta (Quinone reductase)-like 1 | transcript | Cryzl1 | Mus musculus | 2.8728e-10 | 1.13 | 0.89 | 1.27 | 0.79 | 0.93 | 1.08 | 8.05 | 3 | 3 | 3 | 0.08 | 0.03 | 0.12 | 0.8418 | 0.2895 | 0.6232 |
| 3445 | 6 | IB10_L1_M1_H0 | **UniProtKB/Swiss-Prot**: [Q921W4](http://www.uniprot.org/uniprot/Q921W4) [Q921W4-2](http://www.uniprot.org/uniprot/Q921W4-2)   **UniProtKB/TrEMBL**: [D3Z6I4](http://www.uniprot.org/uniprot/D3Z6I4) [D3YZD6](http://www.uniprot.org/uniprot/D3YZD6) [F7BGV1](http://www.uniprot.org/uniprot/F7BGV1) [D3YU21](http://www.uniprot.org/uniprot/D3YU21) | Quinone oxidoreductase-like protein 1, Crystallin, zeta (Quinone reductase)-like 1 | transcript | Cryzl1 | Mus musculus | 2.8728e-10 | 0.65 | 1.53 | 0.76 | 1.32 | 0.89 | 1.12 | 3.45 | 3 | 3 | 3 | 0.10 | 0.15 | 0.15 | 0.0911 | 0.0932 | 0.6661 |
| 1195 | 3 | IB10_L0_M0_H1 | **UniProtKB/Swiss-Prot**: [P35585](http://www.uniprot.org/uniprot/P35585)   **UniProtKB/TrEMBL**: [D3YZ71](http://www.uniprot.org/uniprot/D3YZ71) [S4R1Q4](http://www.uniprot.org/uniprot/S4R1Q4) | AP-1 complex subunit mu-1 | protein | Ap1m1 | Mus musculus | 1.7674e-26 | 1.26 | 0.79 | 1.10 | 0.91 | 1.10 | 0.91 | 9.93 | 3 | 3 | 3 | 0.31 | 0.22 | 0.33 | 0.5238 | 0.7984 | 0.8051 |
| 1195 | 3 | IB10_L1_M1_H0 | **UniProtKB/Swiss-Prot**: [P35585](http://www.uniprot.org/uniprot/P35585)   **UniProtKB/TrEMBL**: [D3YZ71](http://www.uniprot.org/uniprot/D3YZ71) [S4R1Q4](http://www.uniprot.org/uniprot/S4R1Q4) | AP-1 complex subunit mu-1 | protein | Ap1m1 | Mus musculus | 1.7674e-26 | 0.72 | 1.38 | 0.64 | 1.56 | 0.97 | 1.03 | 15.60 | 4 | 4 | 4 | 0.25 | 0.59 | 0.56 | 0.2021 | 0.0091 | 0.9997 |
| **UMTS** | | | | | | | | | | | | | | | | | | | | | | | | |
| **GroupID** | **Size** | **Experiment** | **Protein accessions** | **Protein names** | **Evidence** | **Genes** | **Organism** | **PEP** | **H/L** | **L/H** | **H/M** | **M/H** | **M/L** | **L/M** | **SeqCov** | **Nq HL** | **Nq HM** | **Nq ML** | **SD HL** | **SD HM** | **SD ML** | **SigB HL** | **SigB HM** | **SigB ML** |
| 865 | 4 | VH10_L0_M0_H1 | **UniProtKB/Swiss-Prot**: [O75391](http://www.uniprot.org/uniprot/O75391)   **UniProtKB/TrEMBL**: [I3L0X5](http://www.uniprot.org/uniprot/I3L0X5) [I3L4C3](http://www.uniprot.org/uniprot/I3L4C3) [I3L0Q5](http://www.uniprot.org/uniprot/I3L0Q5) | Sperm-associated antigen 7 | protein | SPAG7 | Homo sapiens | 3.8491e-102 | 1.61 | 0.62 | 1.77 | 0.57 | 0.87 | 1.14 | 15.86 | 2 | 2 | 2 | 0.40 | 0.05 | 0.22 | 0.0959 | 0.0009 | 0.4443 |
| 865 | 4 | VH10_L1_M1_H0 | **UniProtKB/Swiss-Prot**: [O75391](http://www.uniprot.org/uniprot/O75391)   **UniProtKB/TrEMBL**: [I3L0X5](http://www.uniprot.org/uniprot/I3L0X5) [I3L4C3](http://www.uniprot.org/uniprot/I3L4C3) [I3L0Q5](http://www.uniprot.org/uniprot/I3L0Q5) | Sperm-associated antigen 7 | protein | SPAG7 | Homo sapiens | 3.8491e-102 | 0.74 | 1.35 | 0.81 | 1.23 | 1.07 | 0.94 | 10.13 | 2 | 2 | 2 | 0.36 | 0.12 | 0.42 | 0.1447 | 0.1772 | 0.7071 |
| 1018 | 12 | VH10_L0_M0_H1 | **UniProtKB/Swiss-Prot**: [O95825](http://www.uniprot.org/uniprot/O95825) [O95825-2](http://www.uniprot.org/uniprot/O95825-2)   **UniProtKB/TrEMBL**: [A6NMA8](http://www.uniprot.org/uniprot/A6NMA8) [A6NHJ8](http://www.uniprot.org/uniprot/A6NHJ8) [A6NND8](http://www.uniprot.org/uniprot/A6NND8) [C9JZK8](http://www.uniprot.org/uniprot/C9JZK8) [F8WF64](http://www.uniprot.org/uniprot/F8WF64) [C9JAL0](http://www.uniprot.org/uniprot/C9JAL0) [H7C3I5](http://www.uniprot.org/uniprot/H7C3I5) [C9K0F7](http://www.uniprot.org/uniprot/C9K0F7) [C9JQD0](http://www.uniprot.org/uniprot/C9JQD0) [H7C3S0](http://www.uniprot.org/uniprot/H7C3S0) | Quinone oxidoreductase-like protein 1 | protein | CRYZL1 | Homo sapiens | 8.7761e-15 | 0.90 | 1.11 | 0.92 | 1.09 | 0.97 | 1.03 | 4.30 | 2 | 2 | 2 | 0.00 | 0.14 | 0.06 | 0.4018 | 0.4811 | 0.7940 |
| 3872 | 1 | VH10_L0_M0_H1 | **UniProtKB/Swiss-Prot**: [Q9C005](http://www.uniprot.org/uniprot/Q9C005) | Protein dpy-30 homolog | protein | DPY30 | Homo sapiens | 0e+0 | 0.93 | 1.07 | 0.91 | 1.09 | 0.99 | 1.01 | 45.45 | 15 | 15 | 15 | 0.55 | 0.58 | 0.09 | 0.4673 | 0.2093 | 0.9283 |
| 3872 | 1 | VH10_L1_M1_H0 | **UniProtKB/Swiss-Prot**: [Q9C005](http://www.uniprot.org/uniprot/Q9C005) | Protein dpy-30 homolog | protein | DPY30 | Homo sapiens | 0e+0 | 2.12 | 0.47 | 1.94 | 0.52 | 1.04 | 0.96 | 45.45 | 8 | 8 | 8 | 1.12 | 1.17 | 0.06 | 0.0000 | 0.0000 | 0.5529 |
| 3593 | 1 | VH10_L0_M0_H1 | **UniProtKB/Swiss-Prot**: [Q96KP1](http://www.uniprot.org/uniprot/Q96KP1) | Exocyst complex component 2 | protein | EXOC2 | Homo sapiens | 2.6936e-22 | 0.85 | 1.18 | 0.81 | 1.23 | 1.01 | 0.99 | 2.92 | 2 | 2 | 2 | 0.41 | 0.17 | 0.26 | 0.3215 | 0.1770 | 0.9497 |
| 3593 | 1 | VH10_L1_M1_H0 | **UniProtKB/Swiss-Prot**: [Q96KP1](http://www.uniprot.org/uniprot/Q96KP1) | Exocyst complex component 2 | protein | EXOC2 | Homo sapiens | 2.6936e-22 | 2.21 | 0.45 | 2.14 | 0.47 | 1.04 | 0.96 | 2.38 | 2 | 2 | 2 | 1.11 | 0.97 | 0.14 | 0.0000 | 0.0000 | 0.8116 |
| 790 | 4 | VH10_L0_M0_H1 | **UniProtKB/Swiss-Prot**: [O60341](http://www.uniprot.org/uniprot/O60341) [O60341-2](http://www.uniprot.org/uniprot/O60341-2)   **UniProtKB/TrEMBL**: [R4GMP9](http://www.uniprot.org/uniprot/R4GMP9) [R4GMQ1](http://www.uniprot.org/uniprot/R4GMQ1) | Lysine-specific histone demethylase 1A | protein | KDM1A | Homo sapiens | 1.9221e-61 | 0.81 | 1.24 | 0.81 | 1.24 | 0.94 | 1.07 | 6.10 | 4 | 4 | 4 | 1.23 | 0.41 | 1.10 | 0.1035 | 0.1695 | 0.6153 |
| 790 | 4 | VH10_L1_M1_H0 | **UniProtKB/Swiss-Prot**: [O60341](http://www.uniprot.org/uniprot/O60341) [O60341-2](http://www.uniprot.org/uniprot/O60341-2)   **UniProtKB/TrEMBL**: [R4GMP9](http://www.uniprot.org/uniprot/R4GMP9) [R4GMQ1](http://www.uniprot.org/uniprot/R4GMQ1) | Lysine-specific histone demethylase 1A | protein | KDM1A | Homo sapiens | 1.9221e-61 | 1.19 | 0.84 | 1.13 | 0.89 | 1.04 | 0.97 | 8.33 | 6 | 6 | 6 | 0.16 | 0.14 | 0.07 | 0.2729 | 0.4019 | 0.7819 |
| 3593 | 1 | U2OS_L0_M0_H1 | **UniProtKB/Swiss-Prot**: [Q96KP1](http://www.uniprot.org/uniprot/Q96KP1) | Exocyst complex component 2 | protein | EXOC2 | Homo sapiens | 2.6936e-22 | 0.83 | 1.20 | 0.86 | 1.17 | 0.98 | 1.03 | 2.92 | 4 | 4 | 4 | 0.33 | 0.13 | 0.27 | 0.3826 | 0.3650 | 0.9551 |
| 3593 | 1 | U2OS_L1_M1_H0 | **UniProtKB/Swiss-Prot**: [Q96KP1](http://www.uniprot.org/uniprot/Q96KP1) | Exocyst complex component 2 | protein | EXOC2 | Homo sapiens | 2.6936e-22 | 1.55 | 0.65 | 2.06 | 0.49 | 0.85 | 1.17 | 3.25 | 6 | 6 | 6 | 1.10 | 0.97 | 0.20 | 0.0037 | 0.0000 | 0.3112 |
| 858 | 7 | U2OS_L0_M0_H1 | **UniProtKB/Swiss-Prot**: [O75367](http://www.uniprot.org/uniprot/O75367) [O75367-3](http://www.uniprot.org/uniprot/O75367-3) [O75367-2](http://www.uniprot.org/uniprot/O75367-2) [Q9P0M6](http://www.uniprot.org/uniprot/Q9P0M6)   **UniProtKB/TrEMBL**: [B4DJC3](http://www.uniprot.org/uniprot/B4DJC3) [D6RCF2](http://www.uniprot.org/uniprot/D6RCF2) [Q5SQT3](http://www.uniprot.org/uniprot/Q5SQT3) | Core histone macro-H2A.2, Core histone macro-H2A.1, Histone H2A | protein | H2AFY, H2AFY2 | Homo sapiens | 1.9227e-89 | 0.95 | 1.05 | 0.95 | 1.05 | 1.00 | 1.00 | 28.49 | 18 | 18 | 18 | 0.40 | 0.26 | 0.33 | 0.5966 | 0.4114 | 0.8795 |
| 858 | 7 | U2OS_L1_M1_H0 | **UniProtKB/Swiss-Prot**: [O75367](http://www.uniprot.org/uniprot/O75367) [O75367-3](http://www.uniprot.org/uniprot/O75367-3) [O75367-2](http://www.uniprot.org/uniprot/O75367-2) [Q9P0M6](http://www.uniprot.org/uniprot/Q9P0M6)   **UniProtKB/TrEMBL**: [B4DJC3](http://www.uniprot.org/uniprot/B4DJC3) [D6RCF2](http://www.uniprot.org/uniprot/D6RCF2) [Q5SQT3](http://www.uniprot.org/uniprot/Q5SQT3) | Core histone macro-H2A.2, Core histone macro-H2A.1, Histone H2A | protein | H2AFY, H2AFY2 | Homo sapiens | 1.9227e-89 | 1.87 | 0.53 | 1.84 | 0.54 | 0.97 | 1.03 | 23.39 | 11 | 11 | 11 | 0.29 | 0.32 | 0.44 | 0.0000 | 0.0000 | 0.7001 |
| 3604 | 5 | U2OS_L0_M0_H1 | **UniProtKB/Swiss-Prot**: [Q96N66](http://www.uniprot.org/uniprot/Q96N66) [Q96N66-2](http://www.uniprot.org/uniprot/Q96N66-2) [Q96N66-3](http://www.uniprot.org/uniprot/Q96N66-3)   **UniProtKB/TrEMBL**: [A9C4B8](http://www.uniprot.org/uniprot/A9C4B8) [M0R1Z5](http://www.uniprot.org/uniprot/M0R1Z5) | Lysophospholipid acyltransferase 7 | protein | MBOAT7 | Homo sapiens | 3.0432e-14 | 0.64 | 1.56 | 0.77 | 1.29 | 0.88 | 1.14 | 5.08 | 4 | 4 | 4 | 0.64 | 0.97 | 0.42 | 0.0012 | 0.0043 | 0.4166 |
| 3604 | 5 | U2OS_L1_M1_H0 | **UniProtKB/Swiss-Prot**: [Q96N66](http://www.uniprot.org/uniprot/Q96N66) [Q96N66-2](http://www.uniprot.org/uniprot/Q96N66-2) [Q96N66-3](http://www.uniprot.org/uniprot/Q96N66-3)   **UniProtKB/TrEMBL**: [A9C4B8](http://www.uniprot.org/uniprot/A9C4B8) [M0R1Z5](http://www.uniprot.org/uniprot/M0R1Z5) | Lysophospholipid acyltransferase 7 | protein | MBOAT7 | Homo sapiens | 3.0432e-14 | 1.38 | 0.72 | 1.57 | 0.64 | 0.85 | 1.17 | 2.54 | 2 | 2 | 2 | 0.11 | 0.33 | 0.18 | 0.0946 | 0.0203 | 0.3173 |
| 2473 | 4 | U2OS_L0_M0_H1 | **UniProtKB/Swiss-Prot**: [Q13724](http://www.uniprot.org/uniprot/Q13724) [Q13724-2](http://www.uniprot.org/uniprot/Q13724-2)   **UniProtKB/TrEMBL**: [C9J8D4](http://www.uniprot.org/uniprot/C9J8D4) [C9JDQ1](http://www.uniprot.org/uniprot/C9JDQ1) | Mannosyl-oligosaccharide glucosidase | protein | MOGS | Homo sapiens | 2.7399e-16 | 0.79 | 1.26 | 0.89 | 1.12 | 0.94 | 1.06 | 4.90 | 5 | 5 | 5 | 0.56 | 0.64 | 0.18 | 0.1816 | 0.4065 | 0.7958 |
| 2473 | 4 | U2OS_L1_M1_H0 | **UniProtKB/Swiss-Prot**: [Q13724](http://www.uniprot.org/uniprot/Q13724) [Q13724-2](http://www.uniprot.org/uniprot/Q13724-2)   **UniProtKB/TrEMBL**: [C9J8D4](http://www.uniprot.org/uniprot/C9J8D4) [C9JDQ1](http://www.uniprot.org/uniprot/C9JDQ1) | Mannosyl-oligosaccharide glucosidase | protein | MOGS | Homo sapiens | 2.7399e-16 | 1.32 | 0.76 | 1.40 | 0.72 | 0.96 | 1.04 | 5.73 | 4 | 4 | 4 | 0.13 | 0.93 | 1.03 | 0.2943 | 0.0922 | 0.8561 |
| 3706 | 4 | U2OS_L0_M0_H1 | **UniProtKB/Swiss-Prot**: [Q99959](http://www.uniprot.org/uniprot/Q99959) [Q99959-2](http://www.uniprot.org/uniprot/Q99959-2)   **UniProtKB/TrEMBL**: [B8QGS9](http://www.uniprot.org/uniprot/B8QGS9) [A0A087WXY2](http://www.uniprot.org/uniprot/A0A087WXY2) | Plakophilin-2 | protein | PKP2 | Homo sapiens | 9.416e-27 | 0.86 | 1.17 | 0.82 | 1.22 | 0.99 | 1.01 | 6.47 | 8 | 8 | 8 | 0.11 | 0.21 | 0.20 | 0.4055 | 0.1704 | 0.8905 |
| 3706 | 4 | U2OS_L1_M1_H0 | **UniProtKB/Swiss-Prot**: [Q99959](http://www.uniprot.org/uniprot/Q99959) [Q99959-2](http://www.uniprot.org/uniprot/Q99959-2)   **UniProtKB/TrEMBL**: [B8QGS9](http://www.uniprot.org/uniprot/B8QGS9) [A0A087WXY2](http://www.uniprot.org/uniprot/A0A087WXY2) | Plakophilin-2 | protein | PKP2 | Homo sapiens | 9.416e-27 | 1.33 | 0.75 | 1.25 | 0.80 | 1.16 | 0.86 | 6.36 | 5 | 5 | 5 | 0.40 | 0.18 | 0.63 | 0.2747 | 0.2970 | 0.4841 |
| 2740 | 1 | U2OS_L1_M1_H0 | **UniProtKB/Swiss-Prot**: [Q3B726](http://www.uniprot.org/uniprot/Q3B726) | DNA-directed RNA polymerase I subunit RPA43 | protein | TWISTNB | Homo sapiens | 9.369e-18 | 3.25 | 0.31 | 2.23 | 0.45 | 1.45 | 0.69 | 8.28 | 2 | 2 | 2 | 1.26 | 1.04 | 0.25 | 0.0000 | 0.0000 | 0.0678 |
| 3187 | 2 | IB10_L0_M0_H1 | **UniProtKB/Swiss-Prot**: [Q8CGU1](http://www.uniprot.org/uniprot/Q8CGU1)   **UniProtKB/TrEMBL**: [E9Q7U2](http://www.uniprot.org/uniprot/E9Q7U2) | Calcium-binding and coiled-coil domain-containing protein 1 | protein | Calcoco1 | Mus musculus | 1.0162e-9 | 1.29 | 0.78 | 1.47 | 0.68 | 0.95 | 1.05 | 6.08 | 2 | 2 | 2 | 0.08 | 0.10 | 0.07 | 0.1660 | 0.1534 | 0.9120 |
| 3187 | 2 | IB10_L1_M1_H0 | **UniProtKB/Swiss-Prot**: [Q8CGU1](http://www.uniprot.org/uniprot/Q8CGU1)   **UniProtKB/TrEMBL**: [E9Q7U2](http://www.uniprot.org/uniprot/E9Q7U2) | Calcium-binding and coiled-coil domain-containing protein 1 | protein | Calcoco1 | Mus musculus | 1.0162e-9 | 0.78 | 1.29 | 0.76 | 1.32 | 1.10 | 0.91 | 5.35 | 2 | 2 | 2 | 0.24 | 0.22 | 0.11 | 0.2836 | 0.1194 | 0.4489 |
| 699 | 2 | IB10_L0_M0_H1 | **UniProtKB/TrEMBL**: [G3X9Z4](http://www.uniprot.org/uniprot/G3X9Z4) [F6UFZ5](http://www.uniprot.org/uniprot/F6UFZ5) | Protein Pcf11, Cleavage and polyadenylation factor subunit homolog (S. cerevisiae) | predicted | Pcf11 | Mus musculus | 1.8708e-58 | 1.26 | 0.79 | 1.22 | 0.82 | 0.89 | 1.13 | 6.76 | 8 | 8 | 8 | 0.27 | 0.34 | 0.36 | 0.1165 | 0.5396 | 0.7587 |
| 699 | 2 | IB10_L1_M1_H0 | **UniProtKB/TrEMBL**: [G3X9Z4](http://www.uniprot.org/uniprot/G3X9Z4) [F6UFZ5](http://www.uniprot.org/uniprot/F6UFZ5) | Protein Pcf11, Cleavage and polyadenylation factor subunit homolog (S. cerevisiae) | predicted | Pcf11 | Mus musculus | 1.8708e-58 | 0.77 | 1.30 | 0.79 | 1.27 | 0.85 | 1.17 | 1.67 | 2 | 2 | 2 | 0.19 | 0.29 | 0.14 | 0.2672 | 0.1620 | 0.7262 |
| 2706 | 2 | IB10_L0_M0_H1 | **UniProtKB/Swiss-Prot**: [Q80W47](http://www.uniprot.org/uniprot/Q80W47)   **UniProtKB/TrEMBL**: [D3YWK1](http://www.uniprot.org/uniprot/D3YWK1) | WD repeat domain phosphoinositide-interacting protein 2 | protein | Wipi2 | Mus musculus | 1.7143e-156 | 0.92 | 1.09 | 0.92 | 1.09 | 1.02 | 0.98 | 11.91 | 5 | 5 | 5 | 0.19 | 0.14 | 0.08 | 0.6589 | 0.4049 | 0.7133 |
| 2706 | 2 | IB10_L1_M1_H0 | **UniProtKB/Swiss-Prot**: [Q80W47](http://www.uniprot.org/uniprot/Q80W47)   **UniProtKB/TrEMBL**: [D3YWK1](http://www.uniprot.org/uniprot/D3YWK1) | WD repeat domain phosphoinositide-interacting protein 2 | protein | Wipi2 | Mus musculus | 1.7143e-156 | 1.61 | 0.62 | 1.65 | 0.61 | 0.97 | 1.03 | 17.53 | 7 | 7 | 7 | 0.59 | 0.40 | 0.44 | 0.0011 | 0.0064 | 0.9793 |
| 4242 | 3 | IB10_L0_M0_H1 | **UniProtKB/Swiss-Prot**: [Q9D061](http://www.uniprot.org/uniprot/Q9D061) [Q9D061-3](http://www.uniprot.org/uniprot/Q9D061-3) [Q9D061-2](http://www.uniprot.org/uniprot/Q9D061-2) | Acyl-CoA-binding domain-containing protein 6 | transcript | Acbd6 | Mus musculus | 2.2319e-13 | 0.74 | 1.35 | 0.71 | 1.40 | 1.09 | 0.92 | 6.74 | 2 | 2 | 2 | 0.02 | 0.44 | 0.46 | 0.2272 | 0.0492 | 0.5068 |
| 4242 | 3 | IB10_L1_M1_H0 | **UniProtKB/Swiss-Prot**: [Q9D061](http://www.uniprot.org/uniprot/Q9D061) [Q9D061-3](http://www.uniprot.org/uniprot/Q9D061-3) [Q9D061-2](http://www.uniprot.org/uniprot/Q9D061-2) | Acyl-CoA-binding domain-containing protein 6 | transcript | Acbd6 | Mus musculus | 2.2319e-13 | 1.90 | 0.53 | 1.81 | 0.55 | 1.02 | 0.98 | 9.93 | 2 | 2 | 2 | 0.97 | 1.13 | 0.16 | 0.0046 | 0.0141 | 0.6646 |
| **WiFi** | | | | | | | | | | | | | | | | | | | | | | | | |
| **GroupID** | **Size** | **Experiment** | **Protein accessions** | **Protein names** | **Evidence** | **Genes** | **Organism** | **PEP** | **H/L** | **L/H** | **H/M** | **M/H** | **M/L** | **L/M** | **SeqCov** | **Nq HL** | **Nq HM** | **Nq ML** | **SD HL** | **SD HM** | **SD ML** | **SigB HL** | **SigB HM** | **SigB ML** |
| 3520 | 2 | VH10_L0_M0_H1 | **UniProtKB/Swiss-Prot**: [Q8WVC0](http://www.uniprot.org/uniprot/Q8WVC0) [Q8WVC0-2](http://www.uniprot.org/uniprot/Q8WVC0-2) | RNA polymerase-associated protein LEO1 | protein | LEO1 | Homo sapiens | 7.7224e-35 | 0.79 | 1.26 | 0.71 | 1.41 | 1.04 | 0.96 | 3.90 | 3 | 3 | 3 | 0.03 | 0.13 | 0.23 | 0.1747 | 0.0041 | 0.9906 |
| 3520 | 2 | VH10_L1_M1_H0 | **UniProtKB/Swiss-Prot**: [Q8WVC0](http://www.uniprot.org/uniprot/Q8WVC0) [Q8WVC0-2](http://www.uniprot.org/uniprot/Q8WVC0-2) | RNA polymerase-associated protein LEO1 | protein | LEO1 | Homo sapiens | 7.7224e-35 | 1.74 | 0.58 | 1.21 | 0.83 | 1.07 | 0.94 | 5.71 | 4 | 4 | 4 | 0.90 | 0.57 | 0.63 | 0.0842 | 0.3507 | 0.6409 |
| 4428 | 5 | VH10_L0_M0_H1 | **UniProtKB/Swiss-Prot**: [Q9NVS9](http://www.uniprot.org/uniprot/Q9NVS9) [Q9NVS9-3](http://www.uniprot.org/uniprot/Q9NVS9-3) [Q9NVS9-4](http://www.uniprot.org/uniprot/Q9NVS9-4) [Q9NVS9-2](http://www.uniprot.org/uniprot/Q9NVS9-2)   **UniProtKB/TrEMBL**: [J3QQV6](http://www.uniprot.org/uniprot/J3QQV6) | Pyridoxine-5'-phosphate oxidase | protein | PNPO | Homo sapiens | 1.4934e-38 | 0.73 | 1.37 | 0.72 | 1.38 | 1.03 | 0.97 | 30.65 | 6 | 6 | 6 | 0.20 | 0.44 | 0.54 | 0.0392 | 0.0006 | 0.9655 |
| 4428 | 5 | VH10_L1_M1_H0 | **UniProtKB/Swiss-Prot**: [Q9NVS9](http://www.uniprot.org/uniprot/Q9NVS9) [Q9NVS9-3](http://www.uniprot.org/uniprot/Q9NVS9-3) [Q9NVS9-4](http://www.uniprot.org/uniprot/Q9NVS9-4) [Q9NVS9-2](http://www.uniprot.org/uniprot/Q9NVS9-2)   **UniProtKB/TrEMBL**: [J3QQV6](http://www.uniprot.org/uniprot/J3QQV6) | Pyridoxine-5'-phosphate oxidase | protein | PNPO | Homo sapiens | 1.4934e-38 | 1.28 | 0.78 | 1.29 | 0.77 | 1.00 | 1.00 | 11.49 | 2 | 2 | 2 | 0.65 | 0.80 | 0.19 | 0.4896 | 0.3529 | 0.926 |
| 1141 | 2 | VH10_L0_M0_H1 | **UniProtKB/Swiss-Prot**: [P01116](http://www.uniprot.org/uniprot/P01116) [P01116-2](http://www.uniprot.org/uniprot/P01116-2) | GTPase KRas | protein | KRAS | Homo sapiens | 1.0571e-19 | 0.97 | 1.03 | 0.95 | 1.05 | 1.01 | 0.99 | 37.57 | 3 | 3 | 3 | 0.44 | 0.03 | 0.42 | 0.7084 | 0.6689 | 0.8698 |
| 1141 | 2 | VH10_L1_M1_H0 | **UniProtKB/Swiss-Prot**: [P01116](http://www.uniprot.org/uniprot/P01116) [P01116-2](http://www.uniprot.org/uniprot/P01116-2) | GTPase KRas | protein | KRAS | Homo sapiens | 1.0571e-19 | 1.90 | 0.53 | 1.91 | 0.52 | 1.03 | 0.97 | 30.69 | 2 | 2 | 2 | 0.02 | 0.08 | 0.11 | 0.0322 | 0.0641 | 0.8781 |
| 720 | 5 | VH10_L0_M0_H1 | **UniProtKB/Swiss-Prot**: [O15084-1](http://www.uniprot.org/uniprot/O15084-1) [O15084-4](http://www.uniprot.org/uniprot/O15084-4) [O15084](http://www.uniprot.org/uniprot/O15084) [O15084-2](http://www.uniprot.org/uniprot/O15084-2)   **UniProtKB/TrEMBL**: [B4DIW9](http://www.uniprot.org/uniprot/B4DIW9) | Serine/threonine-protein phosphatase 6 regulatory ankyrin repeat subunit A | protein | ANKRD28 | Homo sapiens | 1.856e-28 | 0.88 | 1.14 | 0.81 | 1.23 | 1.06 | 0.94 | 2.85 | 2 | 2 | 2 | 0.22 | 0.26 | 0.00 | 0.4652 | 0.1947 | 0.9731 |
| 4155 | 2 | U2OS_L0_M0_H1 | **UniProtKB/Swiss-Prot**: [Q9H1E3](http://www.uniprot.org/uniprot/Q9H1E3) [Q9H1E3-2](http://www.uniprot.org/uniprot/Q9H1E3-2) | Nuclear ubiquitous casein and cyclin-dependent kinase substrate 1 | protein | NUCKS1 | Homo sapiens | 7.8475e-53 | 0.62 | 1.60 | 0.61 | 1.64 | 1.07 | 0.93 | 10.70 | 4 | 4 | 4 | 1.04 | 0.96 | 0.14 | 0.0002 | 0.0000 | 0.7144 |
| 4155 | 2 | U2OS_L1_M1_H0 | **UniProtKB/Swiss-Prot**: [Q9H1E3](http://www.uniprot.org/uniprot/Q9H1E3) [Q9H1E3-2](http://www.uniprot.org/uniprot/Q9H1E3-2) | Nuclear ubiquitous casein and cyclin-dependent kinase substrate 1 | protein | NUCKS1 | Homo sapiens | 7.8475e-53 | 1.18 | 0.85 | 1.22 | 0.82 | 0.93 | 1.08 | 10.70 | 3 | 3 | 3 | 0.84 | 0.80 | 0.07 | 0.2900 | 0.0079 | 0.3658 |
| 4703 | 6 | U2OS_L0_M0_H1 | **UniProtKB/Swiss-Prot**: [Q9ULX6](http://www.uniprot.org/uniprot/Q9ULX6) [Q9ULX6-2](http://www.uniprot.org/uniprot/Q9ULX6-2)   **UniProtKB/TrEMBL**: [V9GZ50](http://www.uniprot.org/uniprot/V9GZ50) [M0QYT7](http://www.uniprot.org/uniprot/M0QYT7) [M0R010](http://www.uniprot.org/uniprot/M0R010) [M0R1Y5](http://www.uniprot.org/uniprot/M0R1Y5) | A kinase (PRKA) anchor protein 8-like, A-kinase anchor protein 8-like | protein | AKAP8L | Homo sapiens | 2.5455e-22 | 0.69 | 1.46 | 0.82 | 1.22 | 0.84 | 1.19 | 3.56 | 2 | 2 | 2 | 0.42 | 0.52 | 0.15 | 0.0354 | 0.1674 | 0.4205 |
| 4703 | 6 | U2OS_L1_M1_H0 | **UniProtKB/Swiss-Prot**: [Q9ULX6](http://www.uniprot.org/uniprot/Q9ULX6) [Q9ULX6-2](http://www.uniprot.org/uniprot/Q9ULX6-2)   **UniProtKB/TrEMBL**: [V9GZ50](http://www.uniprot.org/uniprot/V9GZ50) [M0QYT7](http://www.uniprot.org/uniprot/M0QYT7) [M0R010](http://www.uniprot.org/uniprot/M0R010) [M0R1Y5](http://www.uniprot.org/uniprot/M0R1Y5) | A kinase (PRKA) anchor protein 8-like, A-kinase anchor protein 8-like | protein | AKAP8L | Homo sapiens | 2.5455e-22 | 1.83 | 0.55 | 1.82 | 0.55 | 1.18 | 0.85 | 3.1 | 2 | 2 | 2 | 0.13 | 0.28 | 0.39 | 0.0273 | 0.0015 | 0.4602 |
| 2038 | 2 | IB10_L0_M0_H1 | **UniProtKB/Swiss-Prot**: [Q5HZJ0](http://www.uniprot.org/uniprot/Q5HZJ0)   **UniProtKB/TrEMBL**: [F6QX82](http://www.uniprot.org/uniprot/F6QX82) | Ribonuclease 3 | protein | Drosha | Mus musculus | 1.4157e-13 | 2.64 | 0.38 | 2.82 | 0.35 | 1.04 | 0.96 | 1.53 | 2 | 2 | 2 | 1.61 | 1.38 | 0.10 | 0.0000 | 0.0000 | 0.7067 |
| 2038 | 2 | IB10_L1_M1_H0 | **UniProtKB/Swiss-Prot**: [Q5HZJ0](http://www.uniprot.org/uniprot/Q5HZJ0)   **UniProtKB/TrEMBL**: [F6QX82](http://www.uniprot.org/uniprot/F6QX82) | Ribonuclease 3 | protein | Drosha | Mus musculus | 1.4157e-13 | 0.65 | 1.55 | 0.49 | 2.02 | 1.33 | 0.75 | 2.04 | 2 | 2 | 2 | 0.86 | 1.37 | 0.52 | 0.0455 | 0.0000 | 0.1388 |
| 2330 | 1 | IB10_L0_M0_H1 | **UniProtKB/Swiss-Prot**: [Q6NXJ0](http://www.uniprot.org/uniprot/Q6NXJ0) | Protein WWC2 | protein | Wwc2 | Mus musculus | 6.6255e-15 | 1.25 | 0.80 | 1.25 | 0.80 | 1.12 | 0.89 | 2.53 | 2 | 2 | 2 | 0.22 | 0.38 | 0.00 | 0.2942 | 0.3283 | 0.4504 |
| 2330 | 1 | IB10_L1_M1_H0 | **UniProtKB/Swiss-Prot**: [Q6NXJ0](http://www.uniprot.org/uniprot/Q6NXJ0) | Protein WWC2 | protein | Wwc2 | Mus musculus | 6.6255e-15 | 0.82 | 1.21 | 0.88 | 1.13 | 0.93 | 1.08 | 6.23 | 6 | 6 | 6 | 0.19 | 0.15 | 0.17 | 0.1862 | 0.1777 | 0.8071 |
| 3783 | 1 | IB10_L0_M0_H1 | **UniProtKB/Swiss-Prot**: [Q9CQE6](http://www.uniprot.org/uniprot/Q9CQE6) | Histone chaperone ASF1A | transcript | Asf1a | Mus musculus | 1.1249e-61 | 1.32 | 0.76 | 1.36 | 0.74 | 1.05 | 0.95 | 12.75 | 4 | 4 | 4 | 0.36 | 0.36 | 0.09 | 0.0545 | 0.0411 | 0.5790 |
| 3783 | 1 | IB10_L1_M1_H0 | **UniProtKB/Swiss-Prot**: [Q9CQE6](http://www.uniprot.org/uniprot/Q9CQE6) | Histone chaperone ASF1A | transcript | Asf1a | Mus musculus | 1.1249e-61 | 0.91 | 1.10 | 0.90 | 1.12 | 1.01 | 0.99 | 25.00 | 6 | 6 | 5 | 0.29 | 0.37 | 0.13 | 0.5224 | 0.2306 | 0.7962 |
| 3993 | 7 | IB10_L0_M0_H1 | **UniProtKB/Swiss-Prot**: [Q9CZA6](http://www.uniprot.org/uniprot/Q9CZA6) [Q9CZA6-2](http://www.uniprot.org/uniprot/Q9CZA6-2) [Q9CZA6-3](http://www.uniprot.org/uniprot/Q9CZA6-3) [Q9CZA6-4](http://www.uniprot.org/uniprot/Q9CZA6-4) [Q9ERR1](http://www.uniprot.org/uniprot/Q9ERR1) [Q9ERR1-2](http://www.uniprot.org/uniprot/Q9ERR1-2)   **UniProtKB/TrEMBL**: [F6Q325](http://www.uniprot.org/uniprot/F6Q325) | Nuclear distribution protein nudE homolog 1, Nuclear distribution protein nudE-like 1 | protein | Nde1, Ndel1 | Mus musculus | 5.6735e-16 | 1.15 | 0.87 | 1.23 | 0.81 | 0.94 | 1.07 | 6.98 | 2 | 2 | 2 | 0.39 | 0.54 | 0.17 | 0.5401 | 0.3646 | 0.8266 |
| 3993 | 7 | IB10_L1_M1_H0 | **UniProtKB/Swiss-Prot**: [Q9CZA6](http://www.uniprot.org/uniprot/Q9CZA6) [Q9CZA6-2](http://www.uniprot.org/uniprot/Q9CZA6-2) [Q9CZA6-3](http://www.uniprot.org/uniprot/Q9CZA6-3) [Q9CZA6-4](http://www.uniprot.org/uniprot/Q9CZA6-4) [Q9ERR1](http://www.uniprot.org/uniprot/Q9ERR1) [Q9ERR1-2](http://www.uniprot.org/uniprot/Q9ERR1-2)   **UniProtKB/TrEMBL**: [F6Q325](http://www.uniprot.org/uniprot/F6Q325) | Nuclear distribution protein nudE homolog 1, Nuclear distribution protein nudE-like 1 | protein | Nde1, Ndel1 | Mus musculus | 5.6735e-16 | 0.66 | 1.51 | 0.69 | 1.44 | 0.95 | 1.06 | 7.85 | 2 | 2 | 2 | 0.25 | 0.11 | 0.14 | 0.0562 | 0.0256 | 0.8990 |
| 2308 | 1 | IB10_L0_M0_H1 | **UniProtKB/Swiss-Prot**: [Q6DID3](http://www.uniprot.org/uniprot/Q6DID3) | Protein SCAF8 | protein | Scaf8 | Mus musculus | 2.0782e-36 | 0.94 | 1.06 | 0.97 | 1.03 | 1.01 | 0.99 | 6.94 | 9 | 9 | 9 | 0.42 | 0.54 | 0.19 | 0.4735 | 0.4757 | 0.8619 |
| 2308 | 1 | IB10_L1_M1_H0 | **UniProtKB/Swiss-Prot**: [Q6DID3](http://www.uniprot.org/uniprot/Q6DID3) | Protein SCAF8 | protein | Scaf8 | Mus musculus | 2.0782e-36 | 1.36 | 0.73 | 1.75 | 0.57 | 0.98 | 1.02 | 6.15 | 7 | 7 | 7 | 0.71 | 0.68 | 0.13 | 0.0306 | 0.0000 | 0.9768 |
| 2943 | 5 | IB10_L0_M0_H1 | **UniProtKB/Swiss-Prot**: [Q8C6P8](http://www.uniprot.org/uniprot/Q8C6P8) [Q8C6P8-2](http://www.uniprot.org/uniprot/Q8C6P8-2)   **UniProtKB/TrEMBL**: [G3UY05](http://www.uniprot.org/uniprot/G3UY05) [G3UYC1](http://www.uniprot.org/uniprot/G3UYC1) [H3BL53](http://www.uniprot.org/uniprot/H3BL53) | Zinc finger protein 57 | protein | Zfp57 | Mus musculus | 1.9874e-26 | 0.82 | 1.22 | 0.76 | 1.32 | 1.05 | 0.95 | 11.40 | 4 | 4 | 4 | 0.32 | 0.13 | 0.24 | 0.1742 | 0.0134 | 0.5726 |
| 2943 | 5 | IB10_L1_M1_H0 | **UniProtKB/Swiss-Prot**: [Q8C6P8](http://www.uniprot.org/uniprot/Q8C6P8) [Q8C6P8-2](http://www.uniprot.org/uniprot/Q8C6P8-2)   **UniProtKB/TrEMBL**: [G3UY05](http://www.uniprot.org/uniprot/G3UY05) [G3UYC1](http://www.uniprot.org/uniprot/G3UYC1) [H3BL53](http://www.uniprot.org/uniprot/H3BL53) | Zinc finger protein 57 | protein | Zfp57 | Mus musculus | 1.9874e-26 | 1.61 | 0.62 | 1.47 | 0.68 | 0.94 | 1.07 | 11.40 | 5 | 5 | 5 | 0.30 | 0.19 | 0.24 | 0.0224 | 0.1155 | 0.8558 |
